# Activity-Dependent Dendritic Spine Shrinkage and Growth Involve Downregulation of Cofilin via Distinct Mechanisms

**DOI:** 10.1371/journal.pone.0094787

**Published:** 2014-04-16

**Authors:** Barbara Calabrese, Jean-Michel Saffin, Shelley Halpain

**Affiliations:** Division of Biological Sciences, and Sanford Consortium for Regenerative Medicine, University of California San Diego, La Jolla, California, United States of America; Western University of Health Sciences, United States of America

## Abstract

A current model posits that cofilin-dependent actin severing negatively impacts dendritic spine volume. Studies suggested that increased cofilin activity underlies activity-dependent spine shrinkage, and that reduced cofilin activity induces activity-dependent spine growth. We suggest instead that both types of structural plasticity correlate with decreased cofilin activity. However, the mechanism of inhibition determines the outcome for spine morphology. RNAi in rat hippocampal cultures demonstrates that cofilin is essential for normal spine maintenance. Cofilin-F-actin binding and filament barbed-end production decrease during the early phase of activity-dependent spine shrinkage; cofilin concentration also decreases. Inhibition of the cathepsin B/L family of proteases prevents both cofilin loss and spine shrinkage. Conversely, during activity-dependent spine growth, LIM kinase stimulates cofilin phosphorylation, which activates phospholipase D-1 to promote actin polymerization. These results implicate novel molecular mechanisms and prompt a revision of the current model for how cofilin functions in activity-dependent structural plasticity.

## Introduction

Mechanisms that regulate the growth and shrinkage of dendritc spines play critical roles in the activity-dependent refinement of circuits during neural development and information storage. Alterations in the actin cytoskeleton of spines underlie such structural changes, and are the subject of intense study [1]. Structural plasticity of dendritic spines has been best characterized at synapses among principle neurons of the neocortex and hippocampus. NMDA receptor-dependent long-term depression (LTD) and long-term potentiation (LTP) of such synapses are usually accompanied by morphological changes in spines. LTD is characterized by dendritic spine shrinkage and reduced F-actin polymerization, in addition to reduced numbers of synaptic AMPA receptors. Conversely, LTP in these neurons is associated with dendritic spine growth and increased F-actin polymerization, in addition to increased numbers of AMPA receptors [2]–[4] Moreover, the actin binding protein cofilin has been implicated in both forms of synaptic structural plasticity [5]–[8].

Two isoforms of cofilin, cofilin-1 and cofilin-2, and the closely related protein known as actin depolymerizing factor (ADF) belong to a small family of actin-binding proteins that we refer to collectively in this paper as “cofilin”, since all three isoforms act in a similar fashion to regulate actin filament turnover [9], [10]_ENREF_7. Cofilin-1 and ADF are expressed at high levels in the adult nervous system; cofilin-2 is present only at relatively low levels [11]. Cofilin-1 and ADF have both been detected in dendritic spines and postsynaptic junctions [12]–[16], as well as in other locations distributed throughout neurons and glial cells [11], [17], [18].

Cofilin is involved in many cellular activities in neuronal and non-neuronal cells. As its best characterized function, cofilin promotes the dynamic turnover of F-actin. Cofilin binds along the sides of actin filaments and induces filament severing [9], [10]. After severing, cofilin remains bound to the pointed end of the newly severed filament and facilitates the removal of the cofilin-bound actin monomer from the pointed end, hence it is often referred to as an “actin depolymerizing factor”. On the other hand, cofilin-mediated filament severing can also promote actin dynamics by generating free barbed ends (FBEs) [19], the preferred sites for F-actin assembly within cells, and/or by ensuring an adequate supply of actin monomer recycled from depolymerizing pointed ends [20]. In neuronal and non-neuronal cells, cofilin activity can drive F-actin dynamics to maintain lamellipodia and create membrane protrusions [21]–[25]. The precise role of cofilin activity in dendritic spines has been less well characterized.

Cofilin activity is regulated by several different mechanisms [9], [10]. Phosphorylation of cofilin on serine 3 (Ser-3) by LIM kinases strongly reduces its F-actin binding and severing activity. Ser-3 phosphorylation is reversed by either of two protein phosphatases, chronophin (CIN) [26] or slingshot (SSH) [27], thereby returning cofilin to its active severing state. Additional mechanisms exist for regulating cofilin activity, and cofilins also are known to carry out cellular functions beyond actin severing [28]. Interestingly, phospho-cofilin itself is not inert, as once thought, and instead can actively stimulate morphological responses in cells via activation of phospholipase D-1 [29], [30].

A widely cited model that has emerged from studies of synaptic structural plasticity in hippocampus posits that spine shrinkage during LTD is mediated by an increase in cofilin activity and that spine expansion during LTP is mediated by suppression of cofilin activity [4]–[6], [8], [31]_ENREF_8. During LTP cofilin phosphorylation on Ser-3 increases in spines [5], [32]. Ser-3 phosphorylation of cofilin during LTP is presumed to suppress the severing of actin filaments, which might otherwise inhibit the net gain in F-actin needed to drive the expansion in spine volume. In a similar fashion, an increase in cofilin-dependent actin severing was proposed to lead to spine shrinkage during LTD [8].

Here, using a well-established pharmacological approach to induce NMDA evoked LTD (nLTD) we explore this model and find, unexpectedly, that several of the key predictions regarding cofilin's role in spine shrinkage are not met. Specifically, we find that constitutive cofilin activity is important for spine maintenance and provide evidence suggesting that the actin severing activity of cofilin is strongly suppressed – not stimulated – during the early phase of nLTD. This loss of cofilin activity mediates spine shrinkage. Moreover, although we confirm that phospho-cofilin is increased during glycine-induced LTP (gLTP), our data indicate that it acts via phospholipase D-1 to induce spine expansion.

## Material and Methods

### Ethics statement

This study was performed in strict accordance with the recommendations in the Guide for the Care and Use of Laboratory Animals of the National Institutes of Health. The Institutional Animal Care and Use Committee (IACUC) of the University of California San Diego specifically approved this study under protocol #S0729. All of the animals were handled according to this approved protocol. All surgical procedures were terminal and anesthesia with isoflurane was used to prevent any suffering.

### Cell culture and transfection

Hippocampal cultures were prepared according to Calabrese and Halpain [33] at a density of 300 cells/mm^2^ and maintained in Neurobasal medium (Gibco), supplemented with B27 (Invitrogen) or with Neurocult SM1 neuronal supplement (STEMCELL Technologies) and 0.5 mM L-glutamine (Sigma). Neurons were transfected at 21 days *in vitro* (DIV) using calcium phosphate precipitation [34]. Cells were incubated with the transfection mixture for 3 h in a 5% CO_2_ incubator at 37°C, washed twice with pre-warmed HBS solution (in mM: 135 NaCl, 4 KCl, 1 Na_2_HPO_4_, 2 CaCl_2_, 1 MgCl_2_, 10 glucose, and 20 HEPES, pH 7.35) and returned to the medium in which they had been growing. Cells were fixed or used for live cell-imaging experiments 1 to 2 days post-transfection.

### nLTD and gLTP induction

nLTD was induced according to Lee et al. [35]. In brief, hippocampal neurons were incubated with 40 µM NMDA (Sigma) added to the culture medium usually for 4 min prior to washing and immediate or delayed (up to 60 min) fixation. In order to investigate cofilin-actin binding during the early stages of spine shrinkage, NMDA was applied for 2 min before cultures were fixed. Glycine-induced LTP (gLTP) was induced according to Fortin et al. [36] by switching the media to a solution that contained the following (in mM): 125 NaCl, 2.5 KCl, 2 CaCl2, 5 HEPES, 33 glucose, 0.2 glycine, 0.02 bicuculline, and 0.003 strychnine for 10 min at room temperature before returning back to 125 NaCl, 2.5 KCl, 2 CaCl2, 1 MgCl2, 5 HEPES; and 33 glucose; pH was adjusted to 7.3 using NaOH. FIPI (Cayman Chemicals) was dissolved in DMSO and used at the final concentration of 0.75 µM.

### cDNA constructs and reagents

pEGFP-N1 and farnesylated eGFP and CFP were obtained from Clontech, pDisplay was a gift from A. Ghosh (UC San Diego), SEP-GluA_2_ was a gift from R. Malinow (UC San Diego), human slingshot (SSH) and LIMK were gifts from the late G.M. Bokoch (The Scripps Research Institute), and Lifeact was a gift from R. Truant (McMaster University, Canada). mcherry was received as pRSETBmcherry (gift of R.Tsien, UC San Diego). To express it in mammalian cells we excised the mcherry containing fragment using the restriction enzyme EcoRI, followed by incubation with the large DNA polymerase fragment Klenow (New England Biolabs) and the restriction enzyme BamH1, and we inserted it into peGFP-N1 back bone after removing eGFP using the restriction enzyme Not I, followed again by incubation with Klenow and BamHI. Rat HA- chronophin (CIN), rat cofilin-HA and rat cofilin shRNA were generated in our laboratory by J. Lauterbach. The CIN cDNA was amplified from female adult rat brain. The primer pairs used for the PCR amplification of rat HA-CIN were: *N-term_HA_Bglll-CIN*, 5′-CGC GGA TCC GCC ACC ATG TAC CCA TAC GAT GTT CCA GAT TAC GCT AGA TCT ATG GCG CGC TGC GAG CGG CTG C-3′; *C-term-EcoRV-CIN* 3′-GGG ATA TCT CAG TCC TCC AGC CCC TCC ATC AAG-5′.

Rat cofilin was amplified from a rat cofilin plasmid kindly provided by J. Birkenfeld (Max-Planck Institute for Brain Research, Frankfurt/Main). The primer pairs used for the PCR amplification of rat cofilin-HA were: *N-term rat CFL*, 5′-CGC GGA TCC GCC ACC ATG GCC TCT GGT GTG GCT GTC TCT G-3′; *C-term rat CFL_PstI-HA-EcoRV*, 3′- GGG ATA TCT CAA GCG TAA TCT GGA ACA TCG TAT GGG TAC TGC AGC AAA GGC TTG CCC TCC AGG GAA ATG-5′. Both DNAs (HA-CIN and Cofilin-HA) were inserted into the pcDNA3.1 plasmid using the BamHI and EcoRV restriction sites. The DNA sequence corresponding to the F3 fragment (aa 585-712) from human PLD1b was amplified by PCR and inserted into the HA-pCDNA3 vector, which was previously cut with EcoRI and dephosphorylated. The primer pairs used for the PCR amplification were: EcoRI_F3-hPLD1_fwd, 5′-ATA-GAA-TTC-ACC-GGG-TCC-ATC-3′; F3-hPLD1_Stop_EcoRI_rev 5′-AGC-GAA-TTC-CTA-ATC-AGC-AGC-3′.

CA074Me was purchased from Calbiochem and used at the final concentration of 4 µM for 30 minutes. FK506 was purchased from Cayman Chemical and used at the final concentration of 1 µM, 1 hr prior to addition of NMDA.

### ADF/Cofilin RNA interference

Rat cofilin shRNA constructs were expressed under control of the polymerase-III H1-RNA gene promoter [37]. The eGFP pSuper vector was that previously used in Calabrese and Halpain [33]. The following oligonucleotides were annealed and inserted into the HindIII/BglII sites of the vector: ADF/cofilin-RNAi, 5′-GAT CCC CGG AGA TTC TTG TAG GAG ATT TCA AGA GAA TCT CCT ACC AGA ATC TCC TTT TTG GAA A-3′, and 5′-AGC TTT TCC AAA AAG GAG ATT CTG GTA GGA GAT TCT CTT GAA ATC TCC TAC CAG AAT CTC CGG G -3′ (corresponding to amino acid sequence EILVGD in ADF, cofilin 1 and 2 rat proteins); non-targeting-RNAi, 5′-GAT CCC CGC GCG CTA TGT AGG ATT CGT TCA AGA GAC GAA TCC TAC ATA GCG CGC TTT TTG GAA A-3′, and 5′-AGC TTT TCC AAA AAG CGC GCT ATG TAG GAT TCG TCT CTT GAA CGA ATC CTA CAT AGC GCG CGG G-3′. Overexpression of cofilin-HA for one day in cultured neurons (1∶1 ratio of cofilin-HA plasmid to pSuper plasmid) was used to evaluate the efficacy of cofilin silencing. The selected plasmid resulted in a complete suppression of cofilin-HA expression in ∼40% of neurons, and a substantial suppression in another 55% of neurons. Other shRNA sequences were also evaluated but were less efficacious in silencing overexpressed cofilin. For knockdown of endogenous ADF/cofilin, neuronal cultures were incubated with the selected shRNA construct for 4 days prior to quantification of spine numbers and morphology. Only those neurons in which both ADF and cofilin 1 immunoreactivity was undetectable in the soma were used for analysis. For these experiments we used the anti-cofilin1 monoclonal antibody MAb22 (kind gift from J.R. Bamburg), which does not cross react with ADF, and the anti-ADF rabbit antibody (1∶100; Sigma, cat #: D8815) previously used to detect selectively ADF (Gorlich et al., 2011).

### Immunocytochemistry

Neurons were fixed with 3.7% formaldehyde in phosphate-buffered saline (PBS) plus 120 mM sucrose for 20 min at 37°C. Neurons were incubated in 20 mM glycine for 5 min, rinsed and permeabilized with 0.2% Triton X-100 for 5 min at room temperature, and then blocked for 30 min with 2% bovine serum albumin (BSA). Chicken anti-total cofilin/ADF antibody AE774 1∶300 (kind gift from J.S. Condeelis), rabbit anti-phosphorylated cofilin/ADF 1∶300 (Abcam, Cambridge, MA; catalog # ab12866), mouse anti-total cofilin/ADF antibody 1∶100 (Abcam, Cambridge, MA; catalog # ab54532), chicken anti-MAP2 antibody 1∶1000 (Lifespan Biosciences), rabbit anti-GFAP antibody 1∶500 (Dako, Denmark), rabbit anti-MAP2 antibody 1∶2000 (S. Halpain), rat anti-HA antibody 1∶500 (Roche Applied Science), rabbit anti-LIMK antibody 5079 1∶500 (kind gift from G.N. Gills), used to detect overexpressed wt-LIMK. All antibodies were incubated for 1 hr at room temperature, and, following rinsing with PBS, were incubated with AlexaFluor-conjugated secondary antibodies (Invitrogen, Molecular Probes) for 45 min at 37°C. To label F-actin, AlexaFluor488-, 568- or 647-phalloidin at 1∶1000 (Invitrogen, Molecular Probes) was incubated for 2 hr at room temperature in the presence of 2% BSA. Finally the coverslips were washed twice with PBS and mounted using Aqua-Mount (Thermo Scientific).

### Time-lapse imaging

Neurons were cultured and transfected on 15 mm borosilicate glass coverslips, which were mounted in a volume of 800 µl at the bottom of a diamond-shaped open bath perfusion chamber, which produces laminar flow enabling fluid exchange times of seconds (Warner Instruments). Live images were acquired every 1–2 minutes over a period of 20–30 minutes with exposure times of 0.01–0.2 s using either an Olympus IX-70 microscope equipped with 405 nm, 491 nm, 561 nm and 640 nm 50 mW solid state lasers (Solamere Technology Group Inc.) and a CoolSNAP HQ2 digital CCD camera (Photometrics) with pixel size of 91 nm, or using a Nikon Ti-E microscope with perfect focus system (Nikon) and a iXon X3 DU897 EM-CCD camera (Andor Technology plc). Both microscopes were equipped with a CSU-X1 spinning disk confocal (Yokogawa Electric Corporation), and a customized CO_2_-delivery, temperature-controlled chamber (5% CO_2_, 35°C). A 60×1.4 NA Plan APO oil immersion objective was used for all the experiments. Only experiments in which focus was perfectly maintained throughout the recording session were included in the time-lapse analyses. Fluorescent specimens were excited using a laser launch (Solamere Technology Group Inc.) equipped with 488 nm, 561 nm and 640 nm 100 mW solid state lasers. Fluorescence emission was selected through the following band-pass filters: 525/50 nm, 595/50, 700/75. Metamorph (Molecular Devices) was used to acquire a stack of images in the z dimension using optical slice thickness of 0.2 or 0.4 µm.

### Quantitative image analysis

In all experiments digital images were acquired using identical parameters and settings (e.g., laser excitation power, acquisition time, time-lapse interval, exposure time, etc.) across experimental conditions. All images displayed in this paper use identical image display settings whenever experimental groups are compared to one another. Spine density, length, and width were analyzed using images displayed in Photoshop (Adobe Systems Inc.) according to Calabrese and Halpain [33]. Most assays were quantified by an observer who was blind to the treatment group identity. Unless otherwise stated, we analyzed a minimum of 15 neurons per treatment group, and replicated the findings using at least 2–3 independent culture preparations; data from different cultures were pooled prior to statistical analysis, as there were no significant differences across preparations. For quantification of spine density, 3–4 dendrite segments totaling at least 150 µm in length per neuron were randomly selected. Sample sizes are provided in all figure legends. Dendrite selection was designed to minimize sampling bias by including approximately equal numbers of dendrites located proximal and distal to the soma (proximal = within 80 µm; distal ≥80 µm). Temporal bias was minimized in time-lapse assays by alternating whether the control or stimulated group was analyzed first in any given imaging session.

#### Quantification of cofilin and phospho-cofilin in nLTD experiments

To quantify the concentration of total cofilin in the dendritic spines and dendritic shafts of cultured hippocampal neurons expressing eGFP we used Image J to digitally subtract the signal derived from astrocytes labeled with the anti-GFAP antibody and create outlines of either the dendritic shaft labeled by MAP2 or dendritic spines highlighted by eGFP. This procedure was developed to provide an objective means of de-selecting regions of dendrites and spines where cofilin signals from surrounding astrocytes would “contaminate” the dendritic cofilin signal. Although this digital procedure typically eliminated less than 15% of the dendritic region defined by the eGFP mask, it was deemed necessary because the degree of astrocytes overlapping with eGFP- transfected dendrites varied considerably among fields of view (Figure S9).

Cell bodies were excluded from the field of view at the time of the acquisition. Cofilin signal was background subtracted before measuring its integrated intensity within either the dendritic spine or dendritic shaft regions. These measurements were normalized by the area of the two dendritic compartments to calculate changes in cofilin concentration.

To quantify relative concentrations of total and phosphorylated cofilin per dendritic spine head within the same neurons we used an alternative approach, because we had only 4 independent wavelengths available for acquisition. ImageJ (National Institutes of Health) was used to create a spatial mask of the dendritic arbor of neurons expressing eGFP as cell filler. We then manually eliminated all irrelevant compartments (dendritic shaft, axons, presynaptic boutons, and spines that were overlying glia or other neurons). The resulting spatial mask of spine heads was multiplied with the images for total or phosphorylated ADF/cofilin to exclude the cofilin signal coming from other compartments. Finally, the concentration of total or phosphorylated ADF/cofilin per spine head was determined by dividing the integrated intensity of the cofilin signal by the surface projection area of the binarized spine heads.

#### Quantification of relative phospho-cofilin concentration in gLTP experiments

To quantify the concentration of phospho-cofilin in dendritic spines in the presence or absence of gLTP we used the Lifeact signal to digitally outline all the spine heads of transfected neurons. The outline was then overlayed onto the background-subtracted image of the endogenous phospho-cofilin signal. Spines overlapping with astrocytes were manually excluded before using Image J to automatically quantify the area of all the spines and the integrated intensity of the phospho-cofilin signal within those same spines.

#### Quantification of SEP-GluA_2_ fluorescence intensity

Images collected at various times for both mcherry (cell filler) and SEP-GluA_2_ were mounted in a single RGB stack and thresholded using ImageJ (National Institutes of Health). Spine area and SEP-GluA_2_ integrated intensity were measured in the same spine at various time points before and after NMDA. The analysis also included the rare instances of spines which contained no detectable SEP-GluA_2_ signal at the beginning of the experiment.

### Free barbed end assay

Alexa Fluor 568–labeled rabbit G-actin was obtained from Invitrogen (A12374) and used to quantify FBEs as described in Shestakova et al.[38], but with the following modifications. G-actin was diluted in 1 mM HEPES pH 7.5, 0.2 mM MgCl_2_, 0.2 mM ATP, sonicated briefly and centrifuged at 4°C at a speed of 50,000 rpm for 10 min using a TLA-100.3 rotor (Beckman). Coverslips containing cultured cells were incubated with 0.85 µM G-actin. Cells were then permeabilized for 90 sec, and maintained at room temperature with the supernatant containing G-actin plus 1% BSA, 0.25 mg/ml saponin, 20 mM HEPES, 138 mM KCl, 4 mM MgCl_2_, 9 mM EGTA and 1 mM ATP, before fixation using 3.7% formaldehyde in PBS. To quantify the concentration of FBEs per dendritic spine the integrated intensity of the G-actin signal was divided by the area of the spine compartment.

### Antibody-based FRET acceptor photobleaching (FRET)

Experiments were performed on a laser scanning microscope (LSM 5 LIVE DuoScan; Carl Zeiss, Inc.) using a 543-nm laser to bleach and excite the acceptor and a 488-nm laser to excite the donor. Acceptor emission was collected using a 560–616 nm band pass filter, and donor emission was collected using a 505–531 nm band pass filter. All imaging was done using 63X NA 1.4 oil objectives. Immunofluorescence was performed using anti-cofilin AE774 (kind gift from J.S. Condeelis) detected using Alexa 488-goat anti–chicken (donor) and anti-β-actin AC15 (Sigma) detected using Alexa 555-goat anti–mouse (acceptor) for the cofilin-actin binding experiments. Based on this method [39], F-actin is fixed, but G-actin is mostly extracted from the cell. Alexa 555, detecting β-actin, was bleached from single dendritic spine heads, and images before and after photobleaching were acquired. The images were analyzed and the FRET efficiency was calculated as E = 1 − (Donor pre/Donor post), according to an algorithm used in the Zeiss LSM 510 FRET AP module of the Zen software; FRET efficiency is expressed as percentage. All images were first background subtracted and corrected for overall sample photobleaching during image collection.

### Duolink Proximity Ligation Assay *in situ* (PLA)

Briefly, cells were fixed according to the protocol used for FRET after 2 min nLTD. After permeabilization with 0.2% Triton X-100 for 10 min cells were incubated in Duolink blocking solution for 30 min at 37°C, then incubated in Duolink Antibody diluent solution at 4°C overnight with the following pairs of primary antibodies: mouse anti-cofilin antibody 1∶100 (Abcam, catalog # ab54532) / rabbit anti-βactin antibody 1∶100 (Novus); rabbit anti-cofilin antibody 1∶200 (Cytoskeleton, Inc. catalog # ACFL02) / mouse anti-βactin AC15 antibody 1∶200 (Sigma); rabbit anti-phosphorylated cofilin antibody 1∶100 (Abcam, catalog # ab12866) / mouse anti-βactin AC15 antibody 1∶200 (Sigma) together with chicken anti-MAP2 antibody 1∶1000 (Lifespan Biosciences). Cells were washed with Duolink buffer A for 5 min with agitation at room temperature. For secondary antibodies conjugated with oligonucleotides, PLA probe anti-mouse MINUS and PLA probe anti-rabbit PLUS were applied in Duolink antibody diluent solution for 2 h at 37°C and washed once again with Duolink buffer A [40]. The following Duolink ligation and amplification steps were performed strictly according to the manufacturer's instructions (www.olink.com). Prior to mounting and imaging, the coverslips were incubated for 30 min using gentle shaking at room temperature with phalloidin 647 1∶500 (Invitrogen), DAPI 1∶1000 (Biotium) and Alexa 488-goat anti-chicken (Invitrogen). Quantification of Duolink puncta was performed using Image J. The MAP2 signal was used to create a binary mask of the entire dendritic arborization within a given image field. This mask was designed to be wider than the actual MAP2 signal, in order to include the region occupied by the spines along those dendrites. Phalloidin was used to identify the dendritic spine region. Image J was used to quantify the number and area of the Duolink puncta included within the binary mask (and these were designated “neuronal Duolink puncta”). To measure total dendritic length per image field, the dendritic mask was reduced to a one-pixel thick line using the skeletonize function in Image J. The number of neuronal Duolink puncta was then normalized by the total dendritic length per image field. The mean area of the Duolink puncta was not found to be significantly different across the various experimental conditions.

### Statistics

All results reported here were observed reproducibly in at least two-three independent culture preparations. A minimum of 15 neurons and 150 micrometers of total dendrite length were sampled per experiment. Prior to quantitative analysis, sample identity was encoded to avoid observer bias. No statistical methods were used to predetermine sample sizes; however, our sample sizes matched or, in most cases, exceeded those generally used in similar studies. Precise sample sizes are stated in the figure legends. Statistical significance was set at the 95% confidence level (two tailed) and calculated using Prism (Graphpad Software). Values are presented as the mean ± S.E.M.

To assess whether the data were normally distributed we used D'Agostin and Pearson test and calculated kurtosis. When normality was not met the appropriate non-parametric test was chosen and described in the legend of the corresponding figure. Variance between groups was taken into account in the selection of the appropriate statistical test. If variances were significantly different we applied Welch's correction. Adjustments for multiple comparisons were made by using one-way ANOVA, two-way ANOVA, or repeated measures ANOVA, as stated in the corresponding figure legends. The selected tests for each figure are defined in the corresponding legends. Pharmacological and genetic experiments were statistically compared to their corresponding vehicle or wild type controls. The experiments that were analyzed in a blind fashion are indicated in the legend of the corresponding figures. The standard error of the ratio of the means (ROM) was calculated using the following function: SE(ROM) = x/y {[(SEMx)^2^/(n_x_ x^2^)]+[(SEMy)^2^/(n_y_ y^2^)]}^½^, where x = mean FRET signal in the presence of NMDA and y =  mean FRET signal in the absence of NMDA.

## Results

### nLTD-induced spine shrinkage and F-actin loss are associated with a decrease in free barbed ends

**Figure 1:**
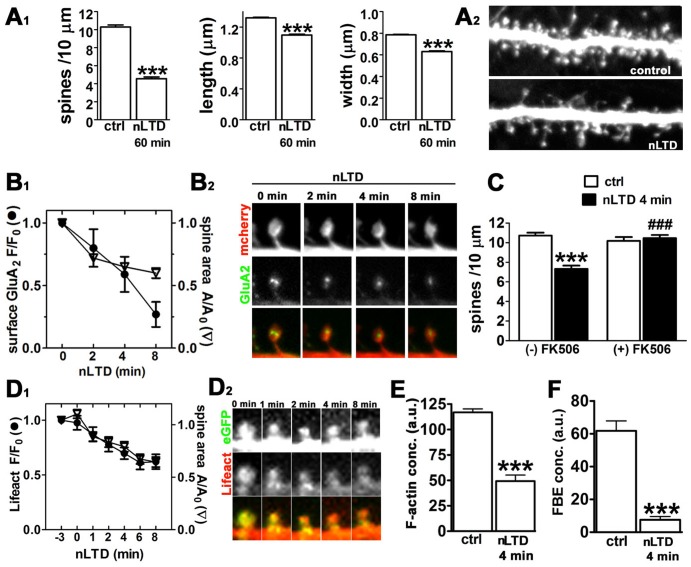
Postsynaptic structural and functional changes induced by NMDA-evoked LTD. Neuronal cultures were prepared and assayed on DIV 21-23, and NMDA-evoked LTD (nLTD) was induced as described in Methods. (**A_1_**) nLTD is associated with long lasting changes on spine density and morphology. Data are expressed as mean ± SEM; ***p<0.0001, Mann-Whitney test (number of dendrites: ctrl = 135, nLTD = 121; number of spines: ctrl = 3753, nLTD = 1490). (**A_2_**) Selected dendritic regions of neurons expressing eGFP illustrate typical spine density and morphology in the absence (*control*) versus presence of NMDA for 4 min (*nLTD*). Image width = 27.3 µm. (**B_1_**) Loss of surface AMPA receptors accompanies the spine shrinkage induced by nLTD. Changes in SEP-GluA_2_ fluorescence (F/F_0_) and spine area (A/A_0_) were quantified using time-lapse imaging during the nLTD stimulus for the indicated times. Data are expressed as mean ± SEM (n = 110 spines). (**B_2_**) Time montage of an individual dendritic spine expressing mcherry and SEP-GluA_2_ at selected time points after nLTD induction (time ‘0’ = start of NMDA addition). Image width = 4.4 µm. (**C**) The calcineurin inhibitor FK506 (1 µM, 1 hr prior to addition of NMDA) reduces nLTD-associated spine loss in eGFP-expressing neurons. Data are expressed as mean ± SEM; ***p<0.001 compared to control alone; ^###^p<0.001 compared to nLTD alone; two-way ANOVA, followed by Bonferroni post hoc test (number of dendrites: ctrl = 56, nLTD_4 min_ = 46, FK506 = 39, nLTD + FK506 = 70). (**D_1_**) nLTD is associated with a decrease in F-actin in live neurons observed using Lifeact. Data are expressed as mean ± SEM; (n = 44 spines). (**D_2_**) Time montage of an individual spine expressing Lifeact-RFP and eGFP at indicated time points following nLTD induction (time ‘0’ = start of NMDA addition). Image width: 3 µm. (**E**) Actin filament concentration is reduced during nLTD. Relative F-actin concentration within spines was quantified as the ratio of the integrated intensity of Alexa568-phalloidin divided by spine area. Data are expressed as mean ± SEM; ***p<0.001, unpaired t-test; a.u.  =  arbitrary units; (number of spines: ctrl = 304, nLTD = 259). (**F**) Free barbed ends of F-actin decrease in spines during nLTD. FBE concentration was quantified as described in Methods. Data are expressed as mean ± SEM; ***p<0.001, unpaired t-test; a.u. = arbitrary units; (number of spines: ctrl = 236, nLTD = 217). Experiments in A, C, E and F and their corresponding supplemental material were analyzed in a blind fashion.

To directly test the hypothesis that activity-induced spine shrinkage involves an increase in F-actin severing by cofilin, we used a pharmacological method for inducing LTD in dissociated cultures of hippocampus [35], which involves a brief application of NMDA to the culture medium (see Material and Methods). Initial experiments showed that lower concentrations of NMDA (20, 30 µM) were able to induce similar spine loss but in a smaller fraction of neurons (data not shown). Experiments also confirmed that a similar magnitude of spine shrinkage occurred when memantine (1 µM) was present to block extra-synaptic NMDA receptors (data not shown) [41]. This NMDA-evoked LTD (nLTD) both occludes synaptically induced LTD and exhibits many of the same key properties and biochemical mechanisms as synaptically induced, NMDA receptor-dependent LTD [35], [42]. Here we confirmed that significant shrinkage and loss of dendritic spines can be detected as early as 2–4 min after the stimulus (Figure 1B) and persist for at least one hour (Figure 1A). Using time-lapse imaging we observed (n = 111 spines over 3 experiments) that within the first 4 min after initiation of nLTD 81% of spines underwent shrinkage, and only 9% of spines expanded by 10% or more (Figure S1 depicts one example experiment). In agreement with other studies using either pharmacological or electrical stimulation to evoke LTD, we also observed that nLTD-induced spine shrinkage was calcineurin dependent (Figure 1C; Figure S2) and accompanied by removal of surface GluA_2_ AMPA receptor subunits (Figure 1B1 and 1B2; Figure S1).

We observed that the nLTD-associated decrease in dendritic spine volume was accompanied by a simultaneous reduction in the concentration of F-actin, as detected either using phalloidin staining in fixed cultures or using Lifeact during time-lapse imaging (Figure 1D1 and 1D2). At 4 min from the initiation of the LTD stimulus the concentration of F-actin was significantly decreased, even after correcting for the reduced spine volume (Figure 1E), in agreement with data showing that F-actin polymerization is inhibited during LTD [43].

If F-actin severing is triggered by LTD, then an increase in the concentration of the free barbed ends (FBEs) of actin filaments should be observed. We tested this prediction using an assay that quantifies FBEs, which involves gently permeabilizing live cells in the presence of fluorescently tagged actin monomer (see Material and Methods). Contrary to prediction, however, we observed an 83% decrease in spine FBE concentration following LTD (Figure1F, Figure S3). This observation appears inconsistent with the hypothesis that LTD activates cofilin-dependent F-actin severing. We therefore sought to address more directly the hypothesis that cofilin's activity is stimulated during LTD.

### nLTD decreases cofilin-actin interaction in spines

Active cofilin binds to F-actin and induces filament severing. Inactive cofilin has a low affinity for F-actin [10], [28]. Therefore, cofilin activity can be assayed by monitoring its binding to F-actin *in situ*. We adopted two different approaches — fluorescence resonance energy transfer (FRET) and Duolink proximity ligation assay (PLA) — to quantify the relative molecular proximity between endogenous cofilin and F-actin within dendritic spines and dendrites at an early time point (2 min) after the initiation of nLTD, while spines are initially shrinking and losing F-actin.

**Figure 2:**
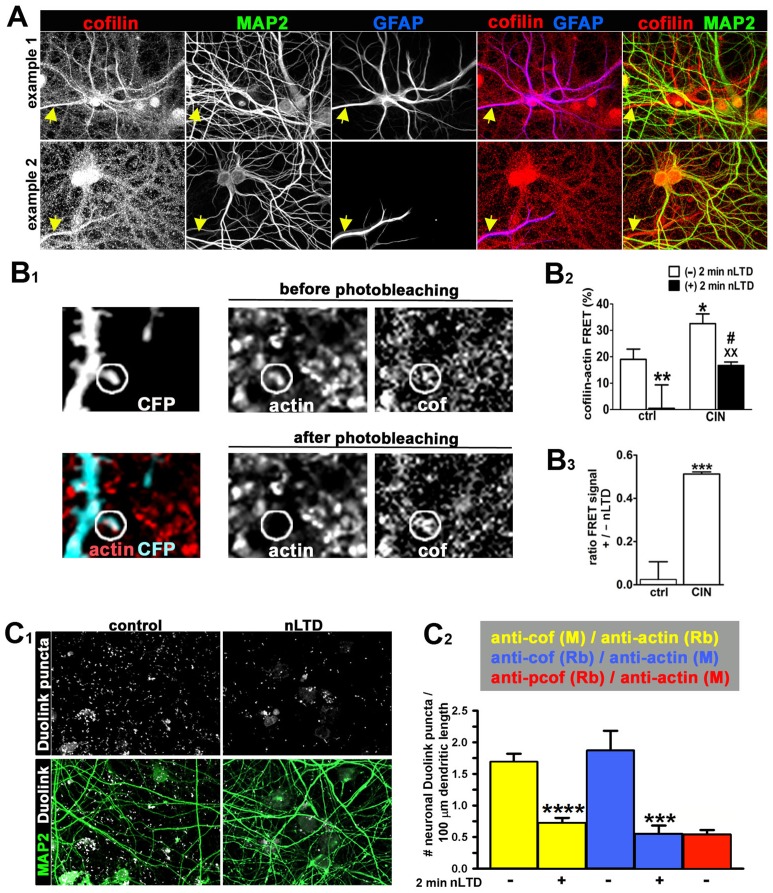
nLTD decreases the molecular proximity between endogenous spine cofilin and F-actin . (**A**) Two examples from control cultures illustrate that endogenous cofilin is present both in neurons (MAP2-positive) and astrocytes (GFAP-positive) *Yellow arrows* point to astrocytic GFAP-positive processes, which often express levels of endogenous cofilin greater than or equal to that in neurons. Image width = 127 µm. (**B**) FRET was carried out as described in Methods. In brief, fixed neurons expressing membrane-targeted CFP were used to select spines for monitoring FRET between fluorescently tagged secondary antibodies against cofilin and actin. (**B_1_**) Example from a typical experiment to illustrate the acceptor photobleaching method. Shown is immunostaining in a control sample for endogenous F-actin and cofilin in a field containing a dendrite expressing membrane targeted CFP (CFP image shown at upper left). Image width = 11 µm. The *white circle* indicates the area that was targeted for acceptor photobleaching. Note that bleaching was restricted to a small region within the actin image, and this same region underwent a corresponding increase in intensity in the cofilin image when the actin is photobleached. For the assay FRET is quantified only within the spine area, not throughout the circular bleached region. (**B_2_**) Quantification of FRET between cofilin and F-actin at dendritic spines of control (ctrl) neurons versus neurons expressing chronophin (CIN) and incubated in the absence or presence of NMDA, as indicated. Data are expressed as mean ± SEM of the “% FRET” values determined using the Zeiss LSM 510 software module for FRET acceptor photobleaching (see Methods); *p<0.05, **p<0.01 compared to control alone; ^#^p<0.05 compared to nLTD alone; ^XX^p<0.01 compared to CIN alone, two-way ANOVA, followed by Bonferroni post hoc test., (number of spines: ctrl = 43, nLTD = 29, CIN = 49, CIN+nLTD = 77). Note that CIN overexpression acted as predicted to significantly increase FRET between endogenous cofilin and F-actin. (**B_3_**) Ratio of cofilin-actin FRET signal with or without the induction of LTD in control neurons versus neurons overexpressing CIN. p<0.001, unpaired t-test with Welch's correction. Data calculated from that in Fig. 2B_2_. (**C_1_**) The *in situ* Duolink proximity ligation assay (PLA) technology was carried out as described in Methods. *White dots* represent the detection of cofilin-actin interaction complexes. Image width = 127 µm. (**C_2_**) Cofilin-actin interaction in control versus nLTD was quantified as the number of neuron-associated Duolink puncta per dendritic length (see Methods). Two different combinations of cofilin and actin primary antibodies were used — one each raised in either rabbit (Rb) or mouse (M), and paired with the appropriate anti-rabbit or anti-mouse secondary antibody. Regardless of antibody combination we found that nLTD decreased the detection of cofilin-actin interaction (data shown in either *blue* or *yellow bars*). Note the low levels for interaction between phospho-cofilin (*p-cof*) and actin (*red bar*), as expected. Data are expressed as mean ± SEM; ****p<0.0001, unpaired t-test, number of fields: [α-cofM/α-actinRb]: 44; ***p<0.001, Mann-Whitney test, number of fields: α-cof Rb/α-actin M = 29; number of fields: α-pcof Rb/α-actin M = 20.

We quantified FRET between endogenous cofilin and endogenous F-actin using fluorescently labeled secondary antibodies to cofilin and actin, as previously described [25]. The fixation method we used selectively extracts monomeric actin while preserving F-actin, and thus reports the relative amount of cofilin that is in close molecular proximity to actin filaments. We quantified FRET using the acceptor photobleaching method [44], and restricted our analysis to dendritic spines, whose area was defined in neurons labeled by transfection with CFP according to methods described [33]. The approach is illustrated in Figure 2B_1_. It was necessary to carefully identify the dendritic spine compartment for these studies because we found not only that cofilin is present in both pre- and postsynaptic compartments, but also that astrocytes display a substantial degree of immunofluorescence for cofilin (Figure 2; two fields of view that include astrocytes are shown in Figure 2A). It was therefore necessary to exclude regions of the sample in which signal from glial cells would obscure signal from the spines. In dendritic spines we observed a significant decrease in the amount of FRET detected at 2 min during nLTD-evoked shrinkage (Figure 2B2).

The FRET assay was validated by observing that neurons overexpressing the cofilin-selective protein phosphatase chronophin (CIN) showed substantially enhanced molecular interaction between cofilin and F-actin (Figure 2B2). CIN stimulates the activity of cofilin via dephosphorylation of Ser-3. CIN overexpression therefore acted as predicted to increase FRET between endogenous cofilin and F-actin. Importantly, we found that CIN overexpression significantly attenuated the ability of nLTD to inhibit the interaction between endogenous cofilin and F-actin (Figure 2B2). Indeed, we observed that the ratio of FRET in the presence versus the absence of nLTD was 2±8% for control neurons and 51±1% for CIN-expressing neurons, a difference that was statistically significant (Figure 2B3). The reduced concentration of F-actin in dendritic spines after nLTD cannot account for this reduced interaction *per se*, since we found no significant correlation (r^2^ = 0.11) between the F-actin staining intensity before photobleaching and the degree of FRET (Figure S5).

As an independent test of whether cofilin-F-actin binding is altered during nLTD, we utilized the DuoLink proximity ligation assay *in situ* [45]. This technique is based on the use of two bifunctional probes, each consisting of a secondary antibody attached to a unique synthetic oligonucleotide that acts as a reporter. A fluorescent signal is generated only when the two target antigens come into sufficient proximity to interact with one another. DNA ligation and polymerization steps provide the amplification necessary to detect single events. We quantified the number of Duolink puncta per image field, and corrected this value for total dendritic length per field (Figure 2C1). To validate the authenticity of this method, we used two different combinations of primary and secondary antibodies each for cofilin and actin, allowing independent experiments using both the anti-mouse and anti-rabbit secondaries for each protein target. Under control conditions the density of Duolink puncta was similar for either combination of primary/secondary antibody pairs, providing confidence that this technology reliably reports molecular proximity. We detected significantly lower density of Duolink puncta using antibodies to actin and phospho-cofilin (Figure 2C2), a result consistent with the expectation that phospho-cofilin should exhibit less molecular proximity to F-actin compared to dephosphorylated cofilin, due to its reduced binding affinity [46]. Importantly, using either antibody combination for the interaction of total cofilin with F-actin, we observed a significant decrease in DuoLink puncta following nLTD (Figure 2C2).

Thus, in both the FRET and PLA assays, we observed a substantial decrease in the molecular proximity (i.e., presumptive binding) between cofilin and F-actin during the initial 2 min of nLTD-evoked spine shrinkage. Together with the results from the free-barbed end assay (Figure 1F), these data strongly suggest that, instead of becoming activated, the filament severing function of cofilin actually becomes inactivated during nLTD.

### Enhancement of cofilin activity prevents spine shrinkage

**Figure 3:**
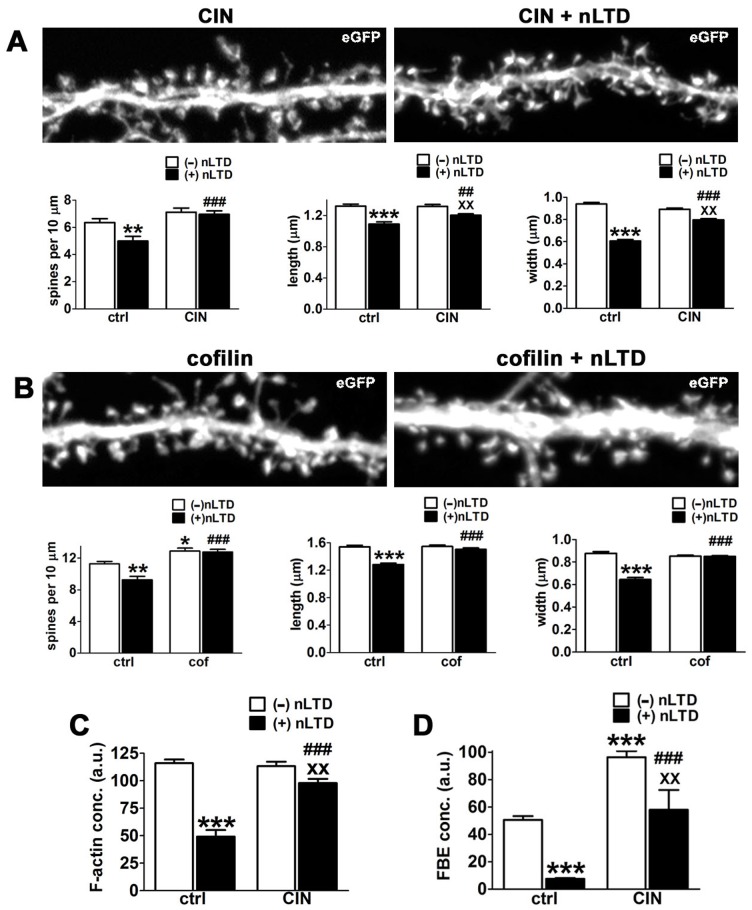
Overexpression of either chronophin or cofilin prevents nLTD-induced loss and shrinkage of dendritic spines. Neurons were transfected with either the cofilin phosphatase chronophin (CIN) or wildtype cofilin itself, as indicated, together with the cell filler eGFP prior to induction of nLTD. Image width = 27.3 µm; all images show eGFP). Morphological data was quantified using eGFP images and are expressed as mean ± SEM; *p<0.05, **p<0.01, ***p<0.001, compared to control alone; ^##^ p<0.01, ^###^p<0.001 compared to nLTD alone; ^XX^p<0.01 compared to CIN or cofilin (cof) alone, two-way ANOVA, followed by Bonferroni post hoc test. (**A**) CIN overexpression prevents nLTD-associated spine loss and shrinkage. Selected dendritic regions of neurons co-expressing CIN and the cell filler eGFP at 4 min post nLTD. For spine density, number of dendrites: ctrl = 38, nLTD = 40, CIN = 32, CIN+nLTD = 59); for spine length and width, number of spines: ctrl = 659, nLTD = 551, CIN = 635, CIN+nLTD = 1109). (**B**) Cofilin overexpression prevents nLTD-associated spine loss and shrinkage. Selected dendritic regions of neurons co-expressing ectopic cofilin and the cell filler eGFP at 4 min post-nLTD. For spine density, number of dendrites: ctrl = 27, nLTD = 21, cof-wt = 54, nLTD+cof-wt = 58); For spine length and width, number of spines: ctrl = 542, nLTD = 348, cof-wt = 1063, nLTD+cof-wt = 1095). (**C**) Chronophin overexpression prevents the nLTD-induced reduction in F-actin concentration in dendritic spines. F-actin concentration in spines was quantified as the ratio of the integrated intensity of Alexa 568-phalloidin divided by spine area on neurons incubated in the absence or presence of NMDA for 4 min (nLTD). number of spines: ctrl = 304, nLTD = 259, CIN = 231, CIN+nLTD = 253; (a.u. = arbitrary units). (**D**) Chronophin overexpression prevents the LTD-induced reduction in actin filament free barbed ends (FBEs) in dendritic spines. FBE concentration was quantified as described in Methods on neurons incubated in the absence or presence of NMDA for 4 min (LTD). Number of spines: ctrl = 236, nLTD = 217, CIN = 96, nLTD+CIN = 167 (a.u. = arbitrary units). Experiments in this figure and its corresponding supplemental material were analyzed in a blind fashion.

We next asked whether stimulating cofilin activity would alter dendritic spine morphology and its changes during nLTD. Contrary to the idea that enhancement of cofilin activity induces dendritic spine shrinkage [8], CIN overexpression by itself had no significant effect on spine numbers or morphology. However, it significantly attenuated the nLTD-associated decrease in spine numbers and spine shrinkage (Figure 3A). Similar results were obtained by overexpression of slingshot, the other known cofilin phosphatase (Figure S6). Protection against nLTD-induced spine shrinkage was also seen with overexpression of exogenous cofilin tagged to hemagglutinin (HA) (Figure 3B). CIN expression was used for subsequent analyses, since we strove to specifically manipulate the endogenous pool of cofilin and minimize potential gain-of-function artifacts induced by cofilin overexpression. Unlike CIN overexpression, cofilin overexpression by itself induced a modest but statistically significant increase in spine numbers (Figure 3B).

In parallel with the prevention of spine shrinkage, overexpression of CIN significantly attenuated the loss in F-actin and FBEs induced during nLTD (Figure 3C and D). In addition CIN induced a statistically significant increase in FBEs, consistent with its role in stimulating cofilin activity (Figure 3D).

### nLTD induces a decrease in total cofilin in spines

We next performed two sets of experiments to address potential mechanisms for the decrease in apparent cofilin activity induced by nLTD. One well documented means of inhibiting cofilin's F-actin severing activity is through phosphorylation on Ser-3 by LIM kinases [9], [10]. We therefore examined whether nLTD might inactivate cofilin by inducing cofilin phosphorylation, or whether a different mechanism predominated.

**Figure 4:**
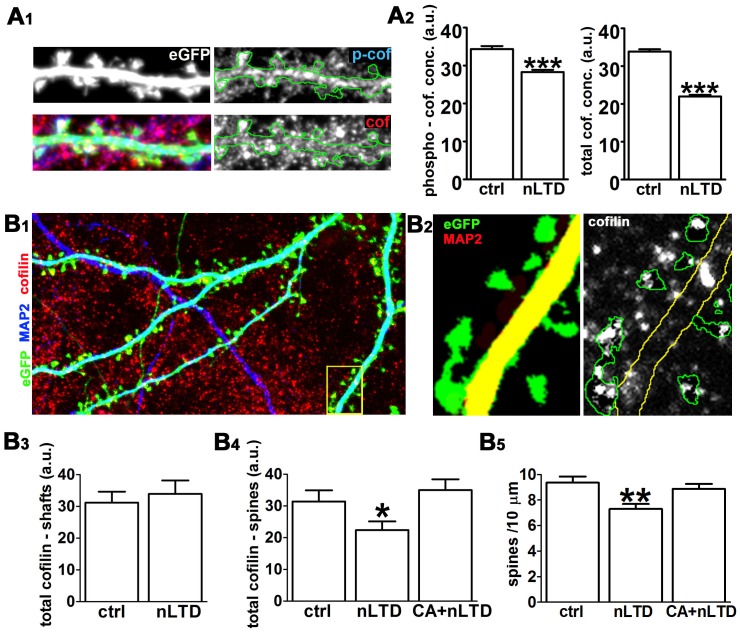
Cofilin concentration selectively decreases in spines following nLTD and involves the capthepsin B/L class of proteases. (**A**) Analysis of total cofilin concentration versus phospho-cofilin concentration in the absence and presence of nLTD. (**A_1_**) Representative example of an eGFP-expressing dendrite costained for phosphorylated (*p-cof*) and total cofilin (*cof*). As described in Methods, a digital processing procedure was used to quantify immunoreactivity only in spine heads (i.e. not the neck or parent dendrite) and also to exclude spines that overlapped with other adjacent structures like astrocytes. At *left* are shown the eGFP image (*top*) and the merged image for eGFP, p-cofilin and total cofilin (*bottom*). At *right* are shown the p-cofilin and total cofilin (*bottom*) images, with the perimeter of the dendrite (*green outline*) overlayed onto each image. Image width = 20 µm. (**A_2_**) Quantification of average phosphorylated (*left*) and total cofilin (*right*) concentrations in dendritic spine heads in the presence or absence of NMDA; integrated immunostaining intensity within each spine was divided by spine area. Data are expressed as mean ± SEM; ***p<0.001; unpaired t-test; number of spines: ctrl = 1608, nLTD = 714; a.u. = arbitrary units. (**B**) Analysis of total cofilin concentration in dendritic spines versus dendritic shafts in the presence and absence of nLTD. (**B_1_**) Cultures were transfected with eGFP prior to being triple-immunostained for MAP2, cofilin/ADF, and GFAP (not shown in the image). The *boxed region* at lower right indicates the region magnified in the two panels in b_2_ at right. Image width = 70 µm. (**B_2_**) Following digital removal of regions stained for GFAP, digital outlines were created based on the eGFP image (*green*) and the MAP2 image (*red* – which, as shown here, appears yellow in the merged image where eGFP and MAP2 are colocalized within the dendritic shaft –). These outlines were then overlaid onto the cofilin image in order to quantify the concentration of cofilin within each subcellular region (see Methods for details. Image width = 11 µm. (**B_3_**) nLTD has no effect on average cofilin concentration in the dendritic shaft. Data are expressed as mean ± SEM; p = 0.9529, Mann-Whitney test number of neurons: ctrl = 26, nLTD = 29. a.u. = arbitrary units. (**B_4_**) nLTD induces a significant decrease in cofilin concentration in dendritic spines that is prevented by incubation with the cathepsin B/L inhibitor CA074Me (4 µM, 30 min). Data are expressed as mean ± SEM; *p<0.05, one-way ANOVA, followed by Tukey post hoc test (number of dendrites: ctrl = 10, nLTD = 25, CA074Me+nLTD = 27). (**B_5_**) The cathepsin inhibitor prevents spine loss during nLTD. **p<0.01 one-way ANOVA followed by Tukey post hoc test (number of neurons: ctrl = 26, nLTD = 29, CA074Me+nLTD = 27). a.u. = arbitrary units. Experiments in this figure and its corresponding supplemental material were analyzed in a blind fashion.

We first characterized the distribution of total cofilin and phospho-cofilin immunoreactivity within the same neurons using, respectively, an antibody that recognizes all three ADF/cofilins independent of phosphorylation state, and a phosphoepitope-specific antibody that recognizes cofilin phosphorylated at Ser-3, which is common to all three isoforms of ADF/cofilin. Immunoreactivity for both antibodies was widely detectable throughout the culture and present in axons, nerve terminals, dendrites, and spines (Figure 4). Immunoreactivity for both antibodies was distributed in a punctate manner. As shown previously (Figure 2A), cofilin concentration in the numerous astrocytes that populate the hippocampal cultures was equal or higher to that in neurons, which would make immunoblot approaches difficult to interpret in a meaningful manner. We therefore endeavored to quantify changes in cofilin levels and phosphorylation state specifically within the dendritic spine compartment. For both sets of experiments described below we observed that the levels of immunoreactivity for cofilin in spines were highly variable and distributed non-uniformly within individual dendritic spines (Figure 4A1, 4B2).

In the first set of experiments, we identified dendritic spines in neurons transfected to express eGFP as a cell-filler. We then quantified the cofilin and phospho-coflin signal only within spines, avoiding dendritic areas that were “contaminated” by cofilin from neighboring astrocytes (see Material and Methods). We observed a modest (∼15%) but reproducible decrease in the concentration of phospho-cofilin in spines (Figure 4A2) at 4 min post-nLTD. However, we also observed a consistent decrease in the concentration of total cofilin of an even greater magnitude (∼30%; Figure 4A2). This suggests that cofilin loss from the spines, rather than increased phosphorylation, might be responsible for much or all of the decreased cofilin activity indicated by the FBE and molecular proximity experiments.

We therefore performed a second series of immunostaining experiments to quantify total cofilin immunoreactivity selectively in either the dendritic spines alone or along the dendritic shaft. This allowed us to probe whether the cofilin loss from spines might be due to proteolysis or to translocation to the dendritic shaft. The dendrites of neurons transfected with eGFP were selected from culture samples that were triple immunostained for cofilin/ADF, MAP2 (to label the dendrites), and GFAP (to label and digitally eliminate the astrocytes; see Material and Methods).

In this second series of experiments we replicated the earlier observation of a 30% decrease in spine cofilin concentration following nLTD (Figure 4B4); we found no statistically significant increase in cofilin concentration in the shaft (Figure 4B3). In addition, we found that CA074Me (4 µM, 30 min), a selective inhibitor of the cathepsin B/L family of proteases blocked the nLTD-induced cofilin loss from dendritic spines (Figure 4B4), and also spine loss (Figure 4B5). The selective proteasome inhibitor MG132 (5 µM for 4 hours) did not prevent nLTD associated cofilin loss or block spine shrinkage (data not shown). Together these results indicate that nLTD stimulates a specific class of proteases that mediate loss of cofilin from dendritic spines, which in turn contributes to spine shrinkage.

### RNAi-mediated silencing of ADF/cofilin induces spine shrinkage

**Figure 5:**
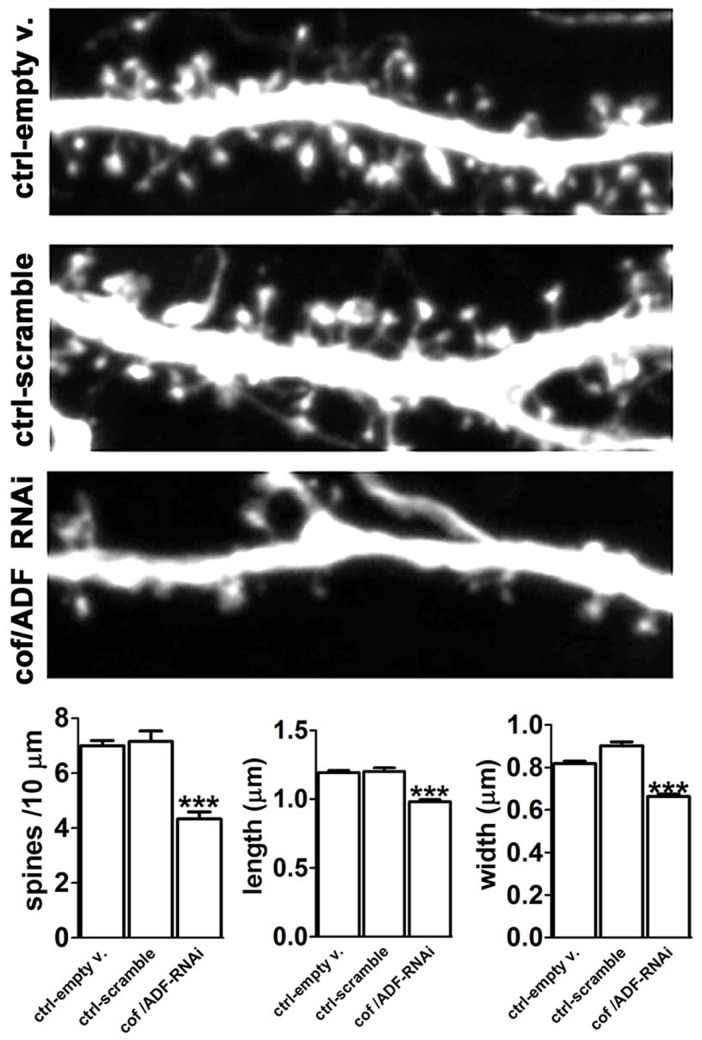
ADF/Cofilin gene silencing induces spine loss and shrinkage. *Upper Panel*: Images from selected dendritic regions of neurons expressing the eGFP-tagged pSuper empty vector, the scrambled control, or the cofilin/ADF-RNAi (cof/ADF-RNAi) for 4 days prior to fixation. Image width = 27.3 µm. *Lower Panel*: Quantification of dendritic spine density, length and width. Data are expressed as mean ± SEM; ***p<0.001, Kruskal-Wallis test followed by Dunn's multiple comparisons test, number of dendrites: empty v.  =  34, scramble = 13, cof/ADF-RNAi = 61; number of spines: empty v. = 642, scramble = 251, cof/ADF-RNAi = 712. Experiments in this figure and its corresponding supplemental material were analyzed in a blind fashion.

The findings described above prompted us to re-evaluate the role of cofilin in spines during resting conditions. Since a previous study reported that ADF levels were increased in cofilin-1 knockout mice [47], we used an shRNA construct designed to silence both ADF and cofilin 1 and 2, to avoid potential gene compensation effects. We introduced this construct for 4 days to 21 DIV neurons that had already established their normal complement of spines, and found that ADF levels were decreased by 48% and cofilin-1 levels were decreased by 91% (Figure S10). The shRNA-mediated suppression of ADF and cofilin-1 expression induced significant spine loss and shrinkage (Figure 5). We therefore conclude that cofilin activity is required to maintain normal spine numbers and morphology. Importantly, these observations are consistent with the hypothesis that loss of cofilin activity during nLTD disrupts the maintenance of spine structure.

### LIMK overexpression induces spine enlargement and prevents spine shrinkage induced by nLTD

**Figure 6:**
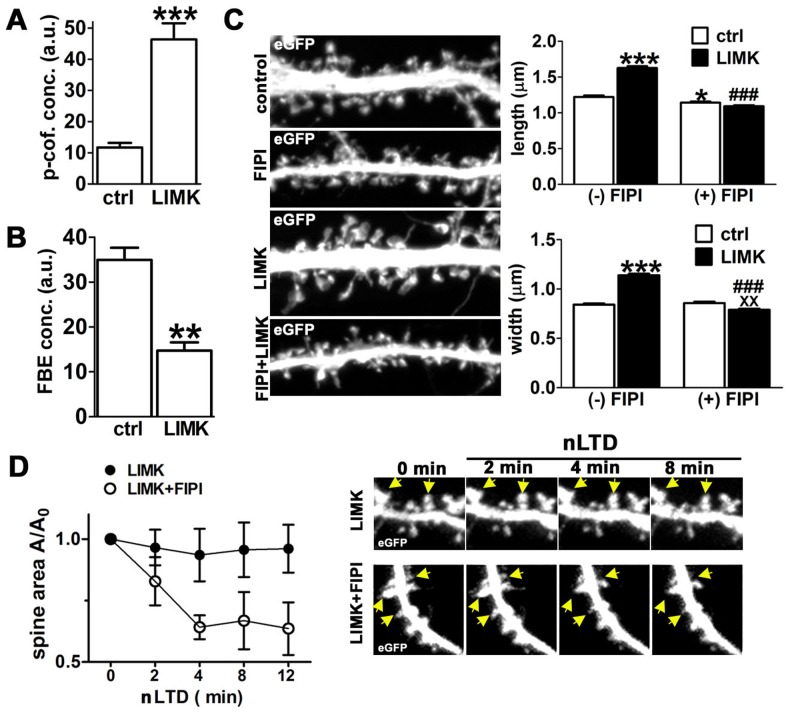
LIMK overexpression induces PLD-dependent spine expansion and prevents nLTD-induced spine shrinkage. (**A**) Quantification of the levels of phospho-cofilin immunoreactivity within dendritic spines in the absence versus presence of overexpressed LIMK for 24 hr. Data are expressed as mean ± SEM; ***p<0.001, unpaired t-test with Welch's correction; number of spines: ctrl = 20 nLTD = 24; a.u. = arbitrary units. (**B**) LIMK overexpression reduces FBEs. Concentration of labeled FBEs in spines of control neurons versus those overexpressing LIMK. Data are expressed as mean ± SEM; **p<0.01, Mann-Whitney test, number of spines: ctrl = 40, nLTD = 153. (**C**) Spine expansion induced by overexpression of LIMK is blocked by the PLD inhibitor FIPI. *Left*: Selected dendritic regions of neurons expressing either eGFP together with mcherry or eGFP together with LIMK for 24 hours while incubated in the presence or absence of FIPI. Only the eGFP channel is shown here. Image width = 27.3 µm. Quantification of spine morphology (length and width) is shown at *right*. Data are expressed as mean ± SEM; number of spines: ctrl = 646, FIPI = 694, LIMK = 830, LIMK+FIPI = 885; * p<0.05, ***p<0.001 compared to control alone; ^###^p<0.001 compared to LIMK alone; ^XX^p<0.01 compared to FIPI alone, two-way ANOVA, followed by Bonferroni post hoc test. Experiments were analyzed in a blind fashion. (**D**) PLD inhibition blocks the prevention of LTD-induced spine shrinkage by LIMK. Changes in spine area (A/A_0_) were quantified using time-lapse imaging of neurons expressing LIMK and eGFP during incubation with NMDA for the indicated times. Data are expressed as mean ± SEM; differences in spine area for the two treatment groups were statistically significant (p = 0.0428) by repeated-measures two-way ANOVA, number of spines: 18. At *right* selected dendritic regions from a time-lapse series during nLTD; *yellow arrows* point to a subset of spines. Image width = 10 µm.

Interestingly, we found that inactivation of cofilin's actin binding activity via LIMK-dependent phosphorylation yielded a different (and seemingly opposite) morphological phenotype for spines as compared to suppression of cofilin through gene silencing. Consistent with previous studies, we found that LIMK overexpression induced a four-fold increase in phospho-cofilin concentration in spines (Figure 6A, Figure S11). As would be expected when cofilin-mediated actin filament severing is inhibited, we also observed a significant reduction in FBE concentration per spine (Figure 6B). However, LIMK overexpression significantly increased spine size (Figure 6C). LIMK overexpression also significantly prevented the spine shrinkage induced by nLTD (Figure 6D).

### Phospholipase D mediates the LIMK effect on spine morphology

Why does dendritic spine morphology react in apparently opposite manners when cofilin-dependent actin severing activity is inhibited via two distinct mechanisms? The striking difference between spine phenotypes induced by cofilin phosphorylation versus cofilin gene silencing suggests that phosphorylated cofilin, while inactive in regard to actin binding and severing activity, might nonetheless induce actin polymerization and spine enlargement. Until recently, phospho-cofilin has been assumed to be functionally inert. However, experiments using non-neuronal cells have demonstrated that Ser-3 phosphorylated cofilin exhibits its own cellular activity via an ability to stimulate phospholipase D-1 (PLD1) [29], [30]. Phospholipase D is known to stimulate F-actin assembly near the plasma membrane [48]-[50].

We therefore evaluated whether the dendritic spine enlargement induced by LIMK might be mediated by phospho-cofilin induced activation of PLD1. Hippocampal neurons were incubated for 24 hours with a PLD blocker, 5-fluoro-2-indolyl des-chlorohalopemide (FIPI, 0.75 µM) [51] immediately following transfection with LIMK cDNA. As shown in Figure 6C, in the presence of this inhibitor dendritic spines failed to enlarge following LIMK overexpression.

In addition, LIMK was no longer able to prevent nLTD-induced spine shrinkage when overexpressed in the presence of FIPI (Figure 6D). Notably, the FIPI inhibitor alone (24 hr) had no effect on spine numbers (data not shown) or spine width, and only slightly but significantly decreased spine length in control neurons. This suggests that PLD is perhaps not highly active under resting conditions, and thus its inhibition *per se* is unlikely to explain the dendritic spine shrinkage induced by nLTD.

### Spine growth during gLTP requires phospholipase D

**Figure 7:**
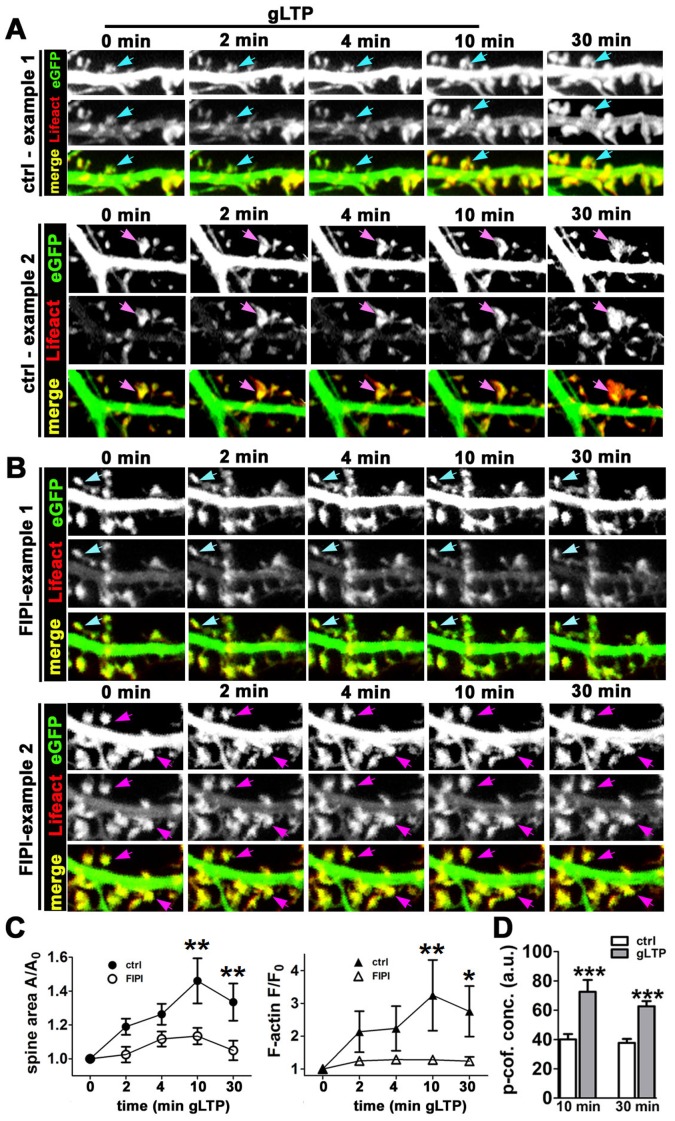
Pharmacological inhibition of PLD prevents gLTP-induced spine enlargement and F-actin polymerization. Time montages of dendritic segments from neurons expressing eGFP and Lifeact incubated in the absence (**A**) or presence (**B**) of the PLD inhibitor FIPI prior to induction of gLTP, as described in Methods. Note the substantial enlargement in dendritic spine size that occurs within 20 min post-gLTP; this expansion is almost completely suppressed by FIPI. Two examples are shown for each condition to illustrate the fact that both small spines and larger spines undergo expansion during gLTP. *Cyan and magenta arrows* indicate, respectively, spines that are small and large before any treatment. Image width = 13 µm. (**C**) *Left*: Quantification of changes in spine size during LTP in the absence versus presence of FIPI, as evaluated using time-lapse images of the cell filler eGFP. Differences in spine area and F-actin intensity for the two treatment groups were statistically significant at 10 and 30 min. Data are expressed as mean ± SEM, **p<0.01, two-way ANOVA, followed by Bonferroni post hoc test, number of spines: ctrl = 39, FIPI = 59. *Right*: Quantification of changes in spine F-actin concentration during LTP in the absence versus presence of FIPI, as evaluated using the Lifeact probe to label endogenous F-actin. Data are expressed as mean ± SEM, *p<0.05, **p<0.01, two-way ANOVA, followed by Bonferroni post hoc test, number of spines: ctrl = 39, FIPI = 59. (**D**) Quantification of average phospho-cofilin concentration in dendritic spines of neurons exposed to control or gLTP conditions for 10 min and then either fixed (10 min) or exchanged back to control buffer solution for an additional 20 minutes before fixation (30 min). Data are expressed as mean ± SEM, ***p<0.001, unpaired t-test, number of neurons (10 min): ctrl = 18, gLTP = 11; ***p<0.001 Mann-Whitney test, number of neurons (30 min): ctrl = 35, gLTP = 42.

Previous studies have demonstrated that hippocampal LTP is associated with an increase in cofilin phosphorylation on Ser-3, as well as a stimulation of signaling pathways upstream of LIMK [5]–[7], [52]. This was proposed to lead to inactivation of cofilin as a key step necessary to promote spine enlargement via new actin polymerization. However, in light of the results described above, it seems possible that during LTP phospho-cofilin might also stimulate actin polymerization via PLD1. We tested this hypothesis in hippocampal cultures using glycine-induced LTP (gLTP) applied to neurons doubly transfected with eGFP as cell filler and Lifeact to label F-actin. In a majority of spines we observed a significant growth in spine volume and increased F-actin intensity within 10 min of gLTP induction; this increase in spine volume and F-actin content persisted for at least 30–60 min, the longest time points examined. Both spine growth and increased F-actin intensity were significantly inhibited in the presence of FIPI (Figure 7A–C). Furthermore, we confirmed that phospho-cofilin concentration was significantly increased in spines at both 10 min and 30 min after induction of gLTP (Figure 7D), in agreement with earlier reports [5], [7].

**Figure 8:**
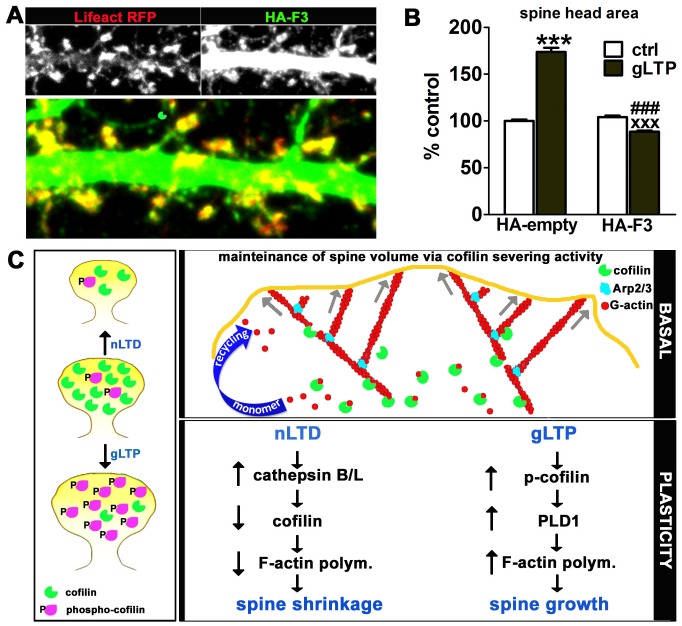
Blocking interaction between phospho-cofilin and PLD1 prevents gLTP-induced spine head enlargement. (**A**) Neuronal cultures were transfected with either empty vector (HA-empty) or HA-tagged F3 fragment of PLD1 (HA-F3), which prevents activation of endogenous PLD1 by endogenous phospho-cofilin, prior to induction of gLTP, as described in Methods. Example images of transfected control neurons are shown in (**A**). Image width = 22.7 µm. Quantification of results (**B**) showed that gLTP induced a significant increase in spine area in the presence of the control construct, but gLTP induced a small but significant decrease in spine area in the presence of the blocking fragment. The fragment by itself had no significant effect on spine area. Data are expressed as mean ± SEM; number of spines (HA-empty vector): ctrl = 3331, gLTP = 3464; number of spines (HA-F3): ctrl = 3608, gLTP = 5377; ***p<0.001 compared to control alone; ^###^p<0.001 compared to gLTP alone; ^XXX^p<0.001 compared to HA-F3 alone, two-way ANOVA, followed by Bonferroni post hoc test. (**C**) Diagram summarizing key findings from this study. The left panel illustrates the observation that dendritic spine shrinkage during nLTD is associated with a decrease in the concentration of cofilin within dendritic spines, while spine growth during gLTP is associated with an increase in the phosphorylation of cofilin on Ser-3, in agreement with previous reports. The upper right panel illustrates our proposed model that under basal conditions cofilin activity is centrally important in maintaining spine volume due to its promotion of actin turnover. Cofilin contributes fresh barbed ends as well as a critical recycling of actin monomer. The mass action of many polymerizing actin filaments provides a steady force against the plasma membrane that helps maintain spine volume. An abrupt loss of cofilin, as during nLTD, leads to a reduction in this internal force. The bottom right panel outlines the signaling pathways that mediate cofilin's role in nLTD-induced spine shrinkage and gLTP-induced spine growth. Experiments in this figure were analyzed in a blind fashion.

Finally, we examined whether phospho-cofilin mediates the activation of PLD1 to induce spine growth during gLTP. We expressed an HA-tagged “F3 fragment” of PLD1, which had been previously shown in non-neuronal cells to specifically block the interaction between phospho-cofilin and PLD1 [29]. Spine expansion in cells expressing the fragment was not only blocked, but showed a statistically significant propensity to shrink following the gLTP stimulus (Figure 8A,B). We therefore conclude that PLD1 activity contributes significantly to spine enlargement during gLTP, and that this effect is mediated by Ser-3 phosphorylated cofilin.

## Discussion

Remodeling of neural circuits consists of changes in synaptic connectivity and synaptic weight, often involving structural changes in the postsynaptic compartment. In many experimental models of activity-dependent plasticity, including cortical and hippocampal LTP and LTD, morphological changes in dendritic spines accompany the insertion and retrieval of glutamate receptors and thus the change in synaptic strength [3], [53], [54]. The actin cytoskeleton is the logical mediator of changes in spine structure, although other factors like membrane trafficking are also likely involved. Actin assembly inhibitors prevent the maintenance of early hippocampal LTP [55], indicating that dynamic actin filaments are essential in this form of synaptic plasticity. Increased actin polymerization in spines is associated with LTP [5], [6], [56], [57] and decreased actin polymerization is associated with LTD [58]. In this study we focused on a role for the actin regulatory protein cofilin as a key player in the early steps of activity-dependent structural plasticity.

Previous studies indicated that during hippocampal LTP cofilin becomes inactivated via Ser-3 phosphorylation to switch off actin filament disassembly, leading to a net gain in actin polymerization and thereby triggering spine expansion [5], [6], [56], [57]. Another study suggested that the opposite mechanism prevails during hippocampal LTD, namely that cofilin becomes activated, leading to a net breakdown of actin filaments and consequent spine shrinkage [8]. Both these models, which have become widely integrated into discussions of synaptic plasticity mechanisms, presume that cofilin activity is inversely correlated with spine volume and spine F-actin concentration.

However, we observed that 1) cofilin/ADF gene silencing via RNAi causes spine loss and shrinkage; 2) FRET and PLA assays indicate that cofilin-F-actin interaction is reduced at early times (2 min) after initiating activity-dependent spine shrinkage; 3) free barbed ends of F-actin are reduced after initiating activity-dependent spine shrinkage; 4) three independent manipulations designed to stimulate intracellular cofilin activity all blocked activity-dependent spine shrinkage, and did not by themselves induce spine shrinkage. None of these results are consistent with the view that cofilin activity negatively impacts spine F-actin or spine volume. In fact, they are all consistent with the opposite view – that cofilin activity maintains spines, and that it is decreased during spine shrinkage.

In addition, although LTP was previously connected to increased levels of phospho-cofilin, which was presumed to suppress cofilin-mediated actin severing [5], [6], [57], it has remained unclear how a reduction in severing alone could account for the requirement of increased actin polymerization during spine growth [55], [56]. Our data suggest that PLD1 represents the missing link between phospho-cofilin and the F-actin-driven spine expansion during LTP.

We therefore propose a new model (Figure 8C) in which constitutive cofilin activity in resting spines is essential to maintain the normal spine volume. It seems likely that the ability of cofilin to “dynamize” actin filaments helps to maintain the high degree of filament turnover present in spines [59], which have polymerizing barbed ends mainly oriented toward the spine membrane [60], [61]. The steady force against the membrane conferred by many polymerizing barbed ends may keep the spine head expanded. We postulate that a decrease in cofilin severing activity disrupts filament dynamics in a manner that results in a net decrease in actin polymerization, thereby weakening the force at the membrane and leading to shrinkage of the spine. On the other hand, activity-dependent spine expansion results from a combination of the suppression in cofilin-mediated F-actin severing and a gain-of-function effect of phospho-cofilin that stimulates PLD1 and leads to increased F-actin polymerization.

In our model, cofilin activity is high under basal conditions and is decreased during either activity-dependent spine growth or activity-dependent spine shrinkage (Figure 8C). However, which biochemical pathway is engaged significantly affects the outcome for spine morphology. Here we observed that activity-dependent spine growth was associated with an increase in cofilin Ser-3 phosphorylation, but activity-dependent spine shrinkage was associated with a significant reduction in cofilin concentration. We postulate that these different means of regulating cofilin activity have distinct consequences for spine actin dynamics, as discussed below.

Our experiments began by testing the hypothesis that stimulation of actin filament severing by cofilin mediates activity-induced loss and shrinkage of spines. Taken together, our results strongly disfavor this prevailing model and question some previous assumptions. First, our data suggest that cofilin severing activity becomes inhibited – not activated – in spines early during nLTD. This is a reasonable interpretation of the observations that, simultaneous with both spine shrinkage and the loss of F-actin, the concentration of free barbed ends of actin filaments dramatically decrease within 4 min of initiating nLTD (Figure 1), and the molecular proximity of cofilin to its severing target F-actin is strongly reduced within 2 min of initiating nLTD, (Figure 2). Although we cannot formally determine the stoichiometry of binding in the intact neuron, it seems likely that the binding we detect between cofilin and F-actin in control conditions represents a severing-competent state of cofilin, rather than a state in which excessive cofilin binding to F-actin inhibits its ability to sever, since the latter only occurs at high concentrations of cofilin *in vitro* [62]. It is therefore reasonable to infer that nLTD induces a decrease in F-actin severing activity by cofilin, since FBEs decrease in concert with reduced cofilin-F-actin interaction.

Second, it has been widely assumed that an increase in cofilin activity necessarily results in a net decrease in spine F-actin and consequent spine shrinkage. Instead, we found that experimentally increasing cofilin activity in neurons, by overexpressing the cofilin phosphatases CIN or slingshot, or by ectopic expression of cofilin itself, did not induce disassembly of the spine actin cytoskeleton or cause spines to shrink (Figure 3). Indeed, such manipulations actually prevented the nLTD-induced loss of F-actin and spine shrinkage.

Finally, the knockdown of cofilin and ADF via gene silencing in mature, three-week old neurons resulted in significant loss and shrinkage of spines (Figure 5). This latter result is not consistent with the view that cofilin-mediated actin severing has a negative impact on spine volume or stability. Instead, it indicates that cofilin is essential for spine maintenance. This may seem surprising given that cofilin-1 knockout mice show increased spine numbers and volume [47]. However, such mice also exhibited substantial increases in ADF concentration; therefore, gene compensation effects may complicate interpretation of those studies. Our cofilin knockdown results are in general agreement with another acute study that reported that in 12 DIV neuronal cultures siRNA specifically against cofilin-1 reduced the numbers of dendritic protrusions [63]. Our data, coupled with this earlier study, indicate that under basal conditions cofilin is needed for both the generation and maintenance of spines, rather than suppression of spine numbers or volume. Nonetheless, there may be specific circumstances in which hyperactivation of cofilin-mediated actin turnover leads to spine shrinkage, and if so further experimental work is required to identify such pathways.

It is worth noting that the results we report here for nLTD are not in conflict with results from a previous paper on synaptically induced LTD [8], although the novel data we obtained here point to a different conclusion regarding cofilin function. In agreement with Zhou et al. (2004), we find that an experimental manipulation designed to increase cofilin Ser-3 phosphorylation prevents spine shrinkage and by itself induces spine enlargement. However, we show here that this effect is dependent upon PLD1 activity, specifically stimulated via phospho-cofilin, a mechanism unknown at the time of the previous work. We furthermore hypothesize that this PLD1-dependent mechanism may also operate during activity-dependent spine growth. We provide evidence in support of this new pathway in experiments using gLTP, where the PLD inhibitor and a PLD1 fragment that prevents phospho-cofilin from binding to PLD1 both inhibit spine enlargement. Our findings do agree with another key conclusion from the Zhou et al (2004) work, however. It appears that the mechanisms for regulating spine shrinkage diverge from those regulating loss of surface AMPA receptors during LTD, since we observe that CIN was able to inhibit spine shrinkage but not loss of SEP-GluA_2_ during nLTD (Figure S4).

We find that both spine shrinkage and spine growth are associated with a reduced concentration of free barbed ends of F-actin in spines. Moreover, both shrinkage and growth are associated with a change in cofilin that should reduce its overall actin severing activity in spines. However, with spine growth there is a net increase in F-actin, while with spine shrinkage there is a net decrease. Thus, the signal transduction cascades that target cofilin in each case must somehow have distinctly different outcomes. During activity-dependent spine growth, the increased phosphorylation of cofilin on Ser-3 is predicted to have at least three potential consequences. First, phospho-cofilin becomes reduced in its actin binding and severing activity. Second, the increased phosphorylation of cofilin will facilitate its release from the actin monomers that depolymerize from pointed ends. Studies of membrane protrusions in non-neuronal cells have proposed that phosphorylation of cofilin by LIMK is important in the local recycling of actin monomers, which are used during actin filament polymerization [20], a factor that might also be critical in dendritic spines (Figure 8C). Third, the activation of PLD1 by phospho-cofilin is predicted to stimulate a burst of actin polymerization, which might synergize with a simultaneous LIMK-driven release of monomer.

Numerous mechanisms have been described for controlling cofilin activity, and it is useful to recognize that cofilin regulation in a cellular context is probably not binary – i.e., not simply switched on and off via phosphorylation. While phospho-cofilin is impaired in actin severing function, dephospho-cofilin is not necessarily active, since it can also become inactivated via other means, including sequestration [19], [28] or, as shown here, through proteolysis. To our knowledge ours is the first study to implicate the cathepsin B/L family of proteases in cofilin loss, and it remains to be determined whether this reflects a direct action of one of these proteases in degrading spine cofilin. Our investigation of cathepsins was prompted by an earlier study where we observed that spine F-actin loss induced by NMDA could be blocked selectively by cathepsin B/L inhibitors but not by inhibitors of the proteasome, calpains, or caspases [64]. An involvement of cathepsin B/L in structural synaptic plasticity is deserving of further attention, as these proteases have been implicated in various aspects of nervous system function and disease [65].

Our observation that endogenous cofilin becomes partially depleted in spines during nLTD contrasts with a recent study reporting that cofilin-GFP accumulates in spines during nLTD [66]. We have observed a similar accumulation of cofilin-GFP (i.e., GFP tagged to the carboxyl terminus of cofilin) in spines of control, transfected neurons in both live and fixed specimens. We find this phenomenon is dramatically increased by induction of nLTD. Cofilin-GFP disappears from the shaft and cell bodies and persists as intensely fluorescent aggregates exclusively in the spine region. In the NMDA condition this was observed 100% of the time in all cofilin-GFP transfected neurons. The accumulation of cofilin-GFP in spines appears as narrow, rod-like aggregates, which seem to resemble small versions of the cofilin-actin rods that accumulate during cellular stress [67], [68]. However, we do not detect this aberrant aggregation with either endogenous cofilin (Figure 4, Figure S7) or with cofilin tagged on the C-terminus with hemagglutinin (Figure S8).

In summary, our data support a fully revised model for the mechanisms that underlie activity-dependent spine shrinkage, and a modification of ideas on the mechanisms that underlie activity-dependent spine growth. We find that a decrease in cofilin levels results in spine shrinkage and F-actin loss, whereas an increase in cofilin phosphorylation, traditionally considered to inactivate cofilin, results in a completely opposite phenotype, i.e., larger spines and more F-actin, due to a gain of function by phospho-cofilin. The critical role for cofilin in spine maintenance that we propose here has implications not only for the development and remodeling of neural circuits, but also for how cofilin may function under pathological conditions, such as Alzheimer's disease, where cofilin mistargeting and abnormal regulation have been observed.

## Supporting Information

Figure S1 nLTD induces spine shrinkage in the majority of dendritic spines. Hippocampal neurons were cultured, co-transfected, and imaged live as described in Methods. Individual spines were monitored for 8–12 minutes by time-lapse imaging for changes in surface AMPA receptors using SEP-GluA_2_ (expressed as the percent change in integrated fluorescence intensity within the spine at t = 4 min versus t = 0 min) and changes in spine size (expressed as percent change in spine area at t = 4 min versus area at t = 0 min) using the cell filler mcherry. Spines were categorized as having ‘shrunk’ if they lost at least 10% of their starting area (*closed triangles*), expanded if they gained at least 10% of their starting area (*closed circles*); or remained unchanged (*open circles*). The majority of spines that shrank also lost surface AMPA receptor. Note, however, that in general the magnitude of changes in surface AMPA receptor were poorly correlated with changes in spine size (R^2^ = 0.03; n = 59).
(JPG)

Figure S2 Inhibition of the protein phosphatase calcineurin attenuates nLTD-induced spine shrinkage. Dendritic spine length and width were quantified in fixed neurons as described in Methods. Data are expressed as mean ± SEM; number of spines: ctrl = 1652, n-LTD = 1075, FK506 = 2222, FK506+nLTD = 911; **p<0.01; ***p<0.001 compared to control alone; ^###^p<0.001 compared to nLTD alone; ^XXX^p<0.001 compared to FK506 alone, two-way ANOVA, followed by Bonferroni post hoc test. Note that calcineurin also prevented the nLTD-induced decrease in spine numbers, as shown in Figure 1C of the main text.
(JPG)

Figure S3 Assay of actin free barbed ends in dendrites of hippocampal neurons. (A) Two examples each of dendritic regions from membrane-targeted eGFP-expressing neurons (GFP-mem) incubated in the absence (*control*) or presence of NMDA for 4 min (*nLTD*). Cultures were fixed and labeled for free barbed ends (FBEs) as described in Methods. Images show the indicated label; for ease of viewing both gray scale and inverted gray scale versions of the FBE channel are provided. Note that FBE puncta are found in most spines in control neurons, but their number, position, and concentration appear to be variable. Following nLTD, spines are fewer in number and remaining spines reduced in area; there is a concomitant reduction in the numbers and size of FBE puncta. For quantification of FBEs as cited in the main text (see Figs. 1, 3, and 6), we quantified the integrated intensity of the FBE signal over the dendritic spines (i.e, excluding the shaft) by using a mask corresponding to the membrane-targeted eGFP dendrite (*eGFP-mem*). Relative FBE concentration was quantified by dividing the FBE signal by the spine area, as determined using the eGFP-mem images (see Methods). Image width = 27 µm. (**B**) Two examples from control cultures are shown at higher magnification to illustrate that many of the FBE puncta are observed within dendritic spine heads. Neurons were triple-labeled for FBEs (*red*); for F-actin using phalloidin (*green*), which is enriched mainly in dendritic spines; and for MAP2 (*blue*), which is enriched in the dendritic shaft. Image width = 7 µm.
(JPG)

Figure S4 Chronophin prevents dendritic spine shrinkage but not loss of surface AMPA receptors associated with nLTD. Loss of surface AMPA receptors accompanies the spine shrinkage induced by nLTD. Changes in SEP-GluA_2_ fluorescence (*left*) and spine area (*right*) were quantified using time-lapse imaging during induction of nLTD for the indicated times. Control neurons display nLTD-induced loss of surface GluA_2_ accompanied by spine shrinkage. Chronophin (CIN) overexpression prevented dendritic spine shrinkage but had no significant effect on the loss of surface GluA_2_. Data are expressed as mean ± SEM; *** indicates significant difference between curves (p<0.001) using two-way ANOVA, followed by Bonferroni post hoc test; number of spines for control neurons = 59 for all time points measured; number of spines for CIN-transfected neurons = 59 for t = 0 min, 2 min, 4 min, but only 35 spines for t = 8 min, due to complete spine collapse for a subset of spines.
(JPG)

Figure S5 FRET efficiency is not significantly correlated with the pre-bleaching intensity of F-actin. Control (*ctrl*) cultures were prepared and immuno-FRET assays performed to quantify the relative molecular proximity between endogenous cofilin and endogenous F-actin within spines, as described in Methods. The graph compares the integrated intensity of the initial actin signal versus the FRET value determined using acceptor photobleaching, as described in Methods (n = 42 spines; R^2^ = 0.11).
(JPG)

Figure S6 Spine loss and shrinkage induced by nLTD are attenuated by slingshot (SSH) overexpression. Neurons were cultured and transfected with eGFP as cell filler alone or together with the coflin phosphatase slingshot (SSH), as described in Methods, prior to incubation in the absence or presence of NMDA for 4 min to induce nLTD. (**A**) Images shown represent the eGFP channel only, for the four different treatment groups: with (+) and without (–) nLTD induction; absence or presence of SSH overexpression. nLTD causes spine loss and shrinkage in eGFP alone (*ctrl*) cultures (*left*) but not in SSH-expressing neurons (*right*). Image width = 27.3 µm. (**B**) Data are expressed as mean ± SEM; for spine density quantification, number of dendrites: ctrl = 22, nLTD (ctrl) = 30, SSH = 25, nLTD (SSH) = 26; for spine length and width quantification, number of spines: ctrl = 527, nLTD (ctrl) = 519, SSH = 578, nLTD (SSH) = 679; **p<0.01; ***p<0.001 compared to control alone; ^###^p<0.001 compared to nLTD alone; ^XXX^p<0.001 compared to SSH alone, two-way ANOVA, followed by Bonferroni post hoc test.
(JPG)

Figure S7 Examples of dendrites stained for endogenous cofilin 4 min post-induction of nLTD. Cofilin immunoreactivity (at *right*) appears weak or absent in most spines relative to control conditions (compare, e.g., to control neuron in Figure 4B2, which was processed in parallel from the same experiment, and is matched for scaling of pixel intensity). In the cofilin images (*right*) the *yellow lines* indicate the outline of the dendritic shaft created from the MAP2 image; and the green lines indicate the outlines of spines created from the eGFP cell filler image (at *left*), after subtraction of the MAP2 image. Image width = 18.
(JPG)

Figure S8 Cofilin-wt-GFP, but not cofilin-wt-HA, redistributes into aberrant aggregates following NMDA addition. Neuronal cultures were transfected with constructs to express ectopic cofilin tagged on the C-terminus with either eGFP or hemaglutinin, as indicated, incubated in the absence or presence of 40 µM NMDA for 4 min (i.e., the ‘nLTD’ condition used throughout this study), and fixed and imaged as described in Methods. HA immunoreactivity was detected using a fluorescently tagged secondary antibody. (**A**) Selected dendritic regions expressing cofilin-wt-HA in the absence (control) or presence of NMDA for 4 min. (**B**) Selected dendritic regions expressing cofilin-wt-GFP in the absence (control) or presence of NMDA. Note the extreme loss of fluorescence within the dendrite shaft and the intense accumulation of fluorescence within rod-like structures, most of which correspond to dendritic spine regions.
(JPG)

Figure S9 Digital processing procedure used to eliminate astrocyte regions from the dendritic region of interest prior to quantification of cofilin immunoreactivity in dendrites and spines. (A) Merged image from a region containing an eGFP expressing dendrite (*green*) adjacent to a GFAP-positive astrocyte (*red*). Note the multiple areas of spatial overlap (*yellow*). Image width = 52 µm. (**B**) In step one, binary masks are created individually from the GFAP image (*left*) and the eGFP image (*right*). (**C**) Next the binarized masks (shown in outline form) are digitally overlaid, and all pixels corresponding the image mask for GFAP (*red outline*) are subtracted from the image mask for eGFP (*cyan outline*), resulting in a dendritic region lacking the areas overlapping with the adjacent astrocytes (**D**). The corresponding outline (**E**) can then be overlaid onto corresponding images of interest, such as cofilin immunoreactivity, in order to quantify signals specifically in dendrites and spines that are free from significant astrocyte-associated signal.
(JPG)

Figure S10 Cofilin/ADF shRNA significantly decreases both cofilin-1 and ADF levels. Neuronal cultures were prepared and shRNA-mediated silencing were carried out as described in Methods. A four day incubation with RNAi construct induced a decrease in cofilin-1 immunoreactivity by approximately 90%, and a decrease in ADF immunoreactivity by approximately 50%. Neither the empty vector nor a scrambled control sequence induced a significant change in immunoreactivity for either cofilin isoform. (**A**) *Left:* Images of a neuron expressing pSuper eGFP scrambled control shRNA, double-stained with antibodies against both cofilin 1 and ADF. *Right*: Images of a “knockdown neuron” expressing pSuper eGFP ADF/cofilin shRNA. The *yellow arrows* indicate cell bodies of transfected neurons, the *cyan arrows* indicate cell bodies of neighboring untransfected neurons. Image width = 127 µm. (**B**) Bar graphs show quantification of the relative concentration of cofilin-1 (cof) or ADF (see Methods for details of the assay). Data represent integrated intensity of the cofilin-1 or ADF signal within the cell soma, and are expressed as mean ± SEM; **p<0.01; ***p<0.001, one-way ANOVA, followed by Tukey's post hoc test; number of cells per condition: empty = 12, scramble = 9, cof/ADF-RNAi = 14. The data are expressed using arbitrary units (a.u.).
(JPG)

Figure S11 LIMK overexpression increases immunoreactivity for endogenous phospho-cofilin in neurons. Selected dendritic regions of neurons expressing either eGFP or eGFP together with LIMK, immunostained for endogenous phosphorylated cofilin (*p-cofilin*). Image width = 35 µm. *Yellow arrows* point to various spines along a region of transfected dendrite; numbered *cyan square boxes* refer to subregions of the dendrite shown at higher magnification in the *cyan-boxed insets*, in which a *yellow outline* of single dendritic spines is overlaid onto the image for phospho-cofilin immunoreactivity. Note that phospho-cofilin immunoreactivity is very low in control neurons (both eGFP-transfected and untransfected). Much of the immunoreactivity seen in a given field of view is attributable to astrocytes. Overexpression of LIMK (*right*) induces a substantial increase in immunoreactivity for phospho-cofilin in neurons, including within spines, which are now readily detectable above the signal from surrounding untransfected neurons and astrocytes.
(JPG)
