# Supplementary figures and images for: Activity-Dependent Dendritic Spine Shrinkage and Growth Involve Downregulation of Cofilin via Distinct Mechanisms
Source: PLoS One. 2014 Apr 16;9(4):e94787. doi: 10.1371/journal.pone.0094787 (PMC3989342; doi:10.1371/journal.pone.0094787)

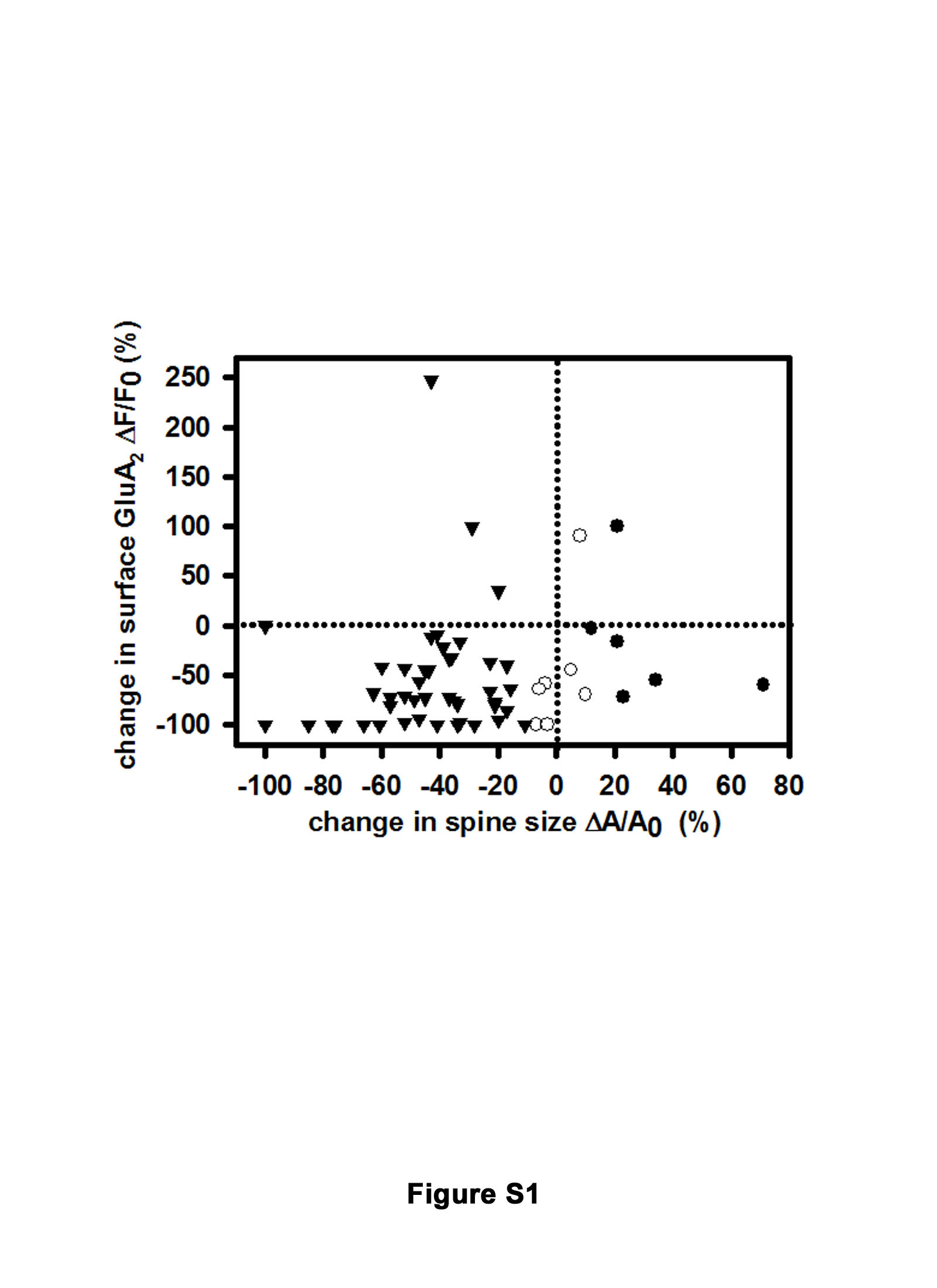

Supplement: Figure S1 — nLTD induces spine shrinkage in the majority of dendritic spines. Hippocampal neurons were cultured, co-transfected, and imaged live as described in Methods. Individual spines were monitored for 8–12 minutes by time-lapse imaging for changes in surface AMPA receptors using SEP-GluA2 (expressed as the percent change in integrated fluorescence intensity within the spine at t = 4 min versus t = 0 min) and changes in spine size (expressed as percent change in spine area at t = 4 min versus area at t = 0 min) using the cell filler mcherry. Spines were categorized as having ‘shrunk’ if they lost at least 10% of their starting area (closed triangles), expanded if they gained at least 10% of their starting area (closed circles); or remained unchanged (open circles). The majority of spines that shrank also lost surface AMPA receptor. Note, however, that in general the magnitude of changes in surface AMPA receptor were poorly correlated with changes in spine size (R2 = 0.03; n = 59). (JPG) [file pone.0094787.s001.jpg]

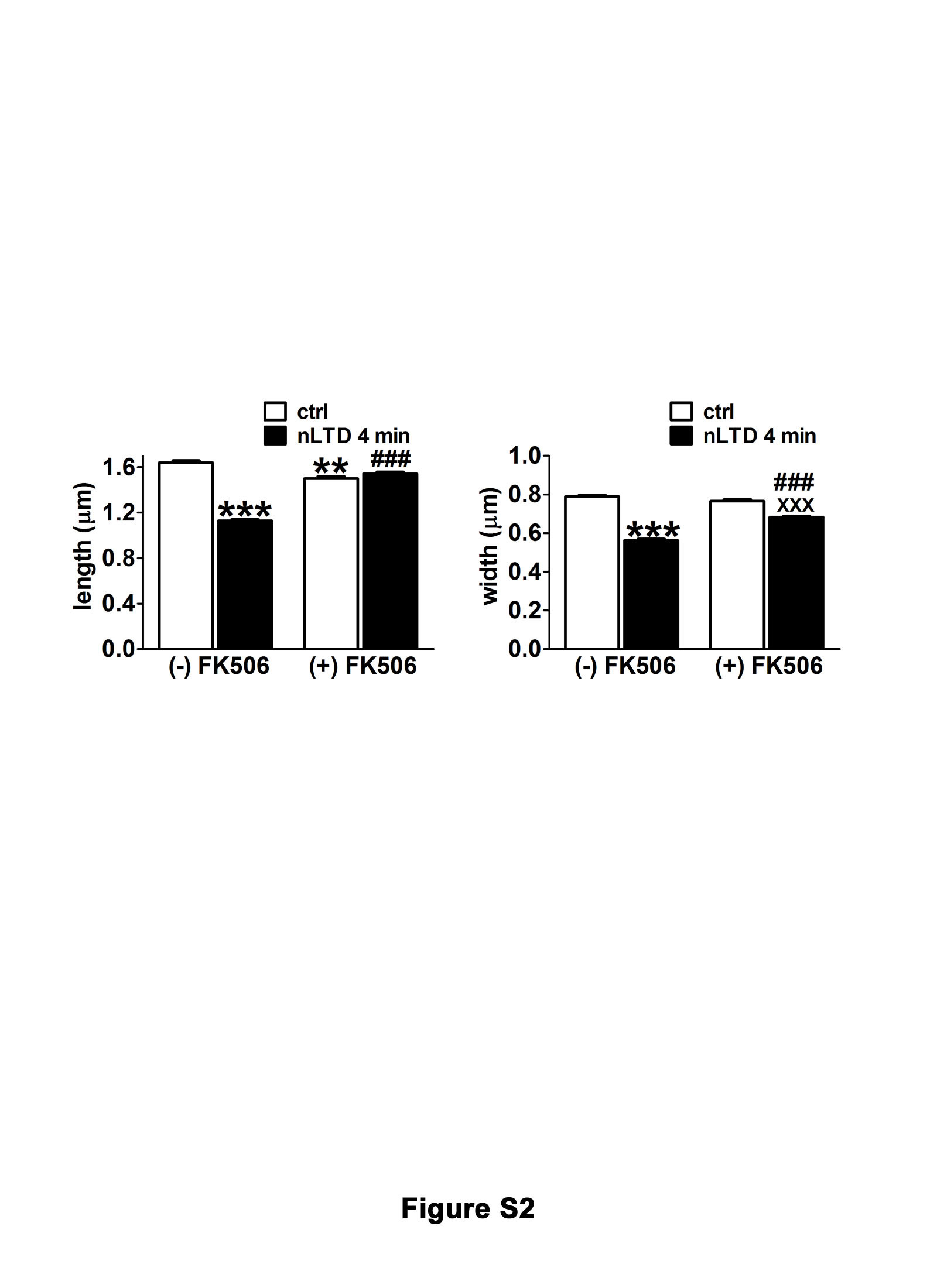

Supplement: Figure S2 — Inhibition of the protein phosphatase calcineurin attenuates nLTD-induced spine shrinkage. Dendritic spine length and width were quantified in fixed neurons as described in Methods. Data are expressed as mean ± SEM; number of spines: ctrl = 1652, n-LTD = 1075, FK506 = 2222, FK506+nLTD = 911; **p<0.01; ***p<0.001 compared to control alone; ###p<0.001 compared to nLTD alone; XXXp<0.001 compared to FK506 alone, two-way ANOVA, followed by Bonferroni post hoc test. Note that calcineurin also prevented the nLTD-induced decrease in spine numbers, as shown in Figure 1C of the main text. (JPG) [file pone.0094787.s002.jpg]

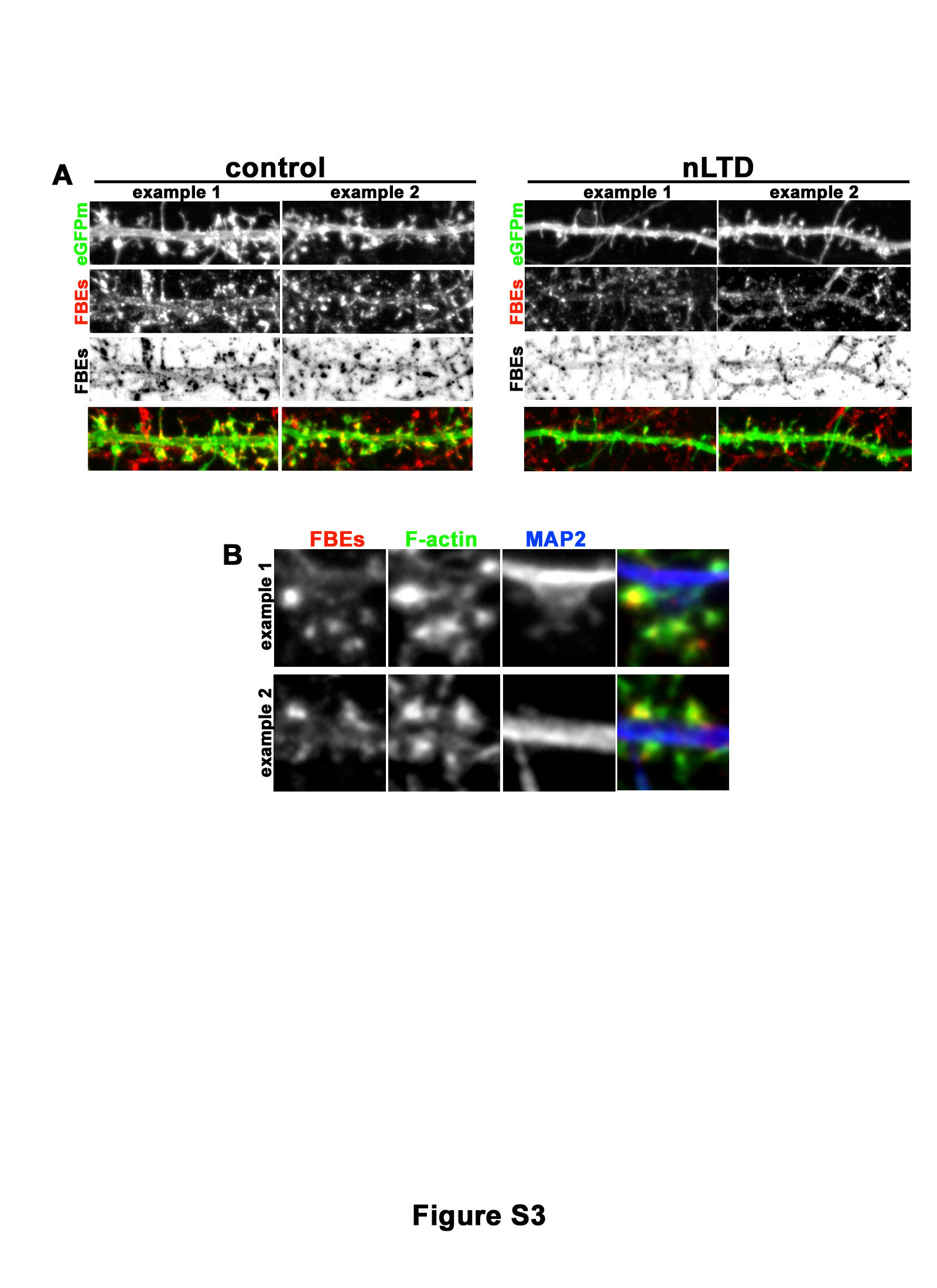

Supplement: Figure S3 — Assay of actin free barbed ends in dendrites of hippocampal neurons. (A) Two examples each of dendritic regions from membrane-targeted eGFP-expressing neurons (GFP-mem) incubated in the absence (control) or presence of NMDA for 4 min (nLTD). Cultures were fixed and labeled for free barbed ends (FBEs) as described in Methods. Images show the indicated label; for ease of viewing both gray scale and inverted gray scale versions of the FBE channel are provided. Note that FBE puncta are found in most spines in control neurons, but their number, position, and concentration appear to be variable. Following nLTD, spines are fewer in number and remaining spines reduced in area; there is a concomitant reduction in the numbers and size of FBE puncta. For quantification of FBEs as cited in the main text (see Figs. 1, 3, and 6), we quantified the integrated intensity of the FBE signal over the dendritic spines (i.e, excluding the shaft) by using a mask corresponding to the membrane-targeted eGFP dendrite (eGFP-mem). Relative FBE concentration was quantified by dividing the FBE signal by the spine area, as determined using the eGFP-mem images (see Methods). Image width = 27 µm. (B) Two examples from control cultures are shown at higher magnification to illustrate that many of the FBE puncta are observed within dendritic spine heads. Neurons were triple-labeled for FBEs (red); for F-actin using phalloidin (green), which is enriched mainly in dendritic spines; and for MAP2 (blue), which is enriched in the dendritic shaft. Image width = 7 µm. (JPG) [file pone.0094787.s003.jpg]

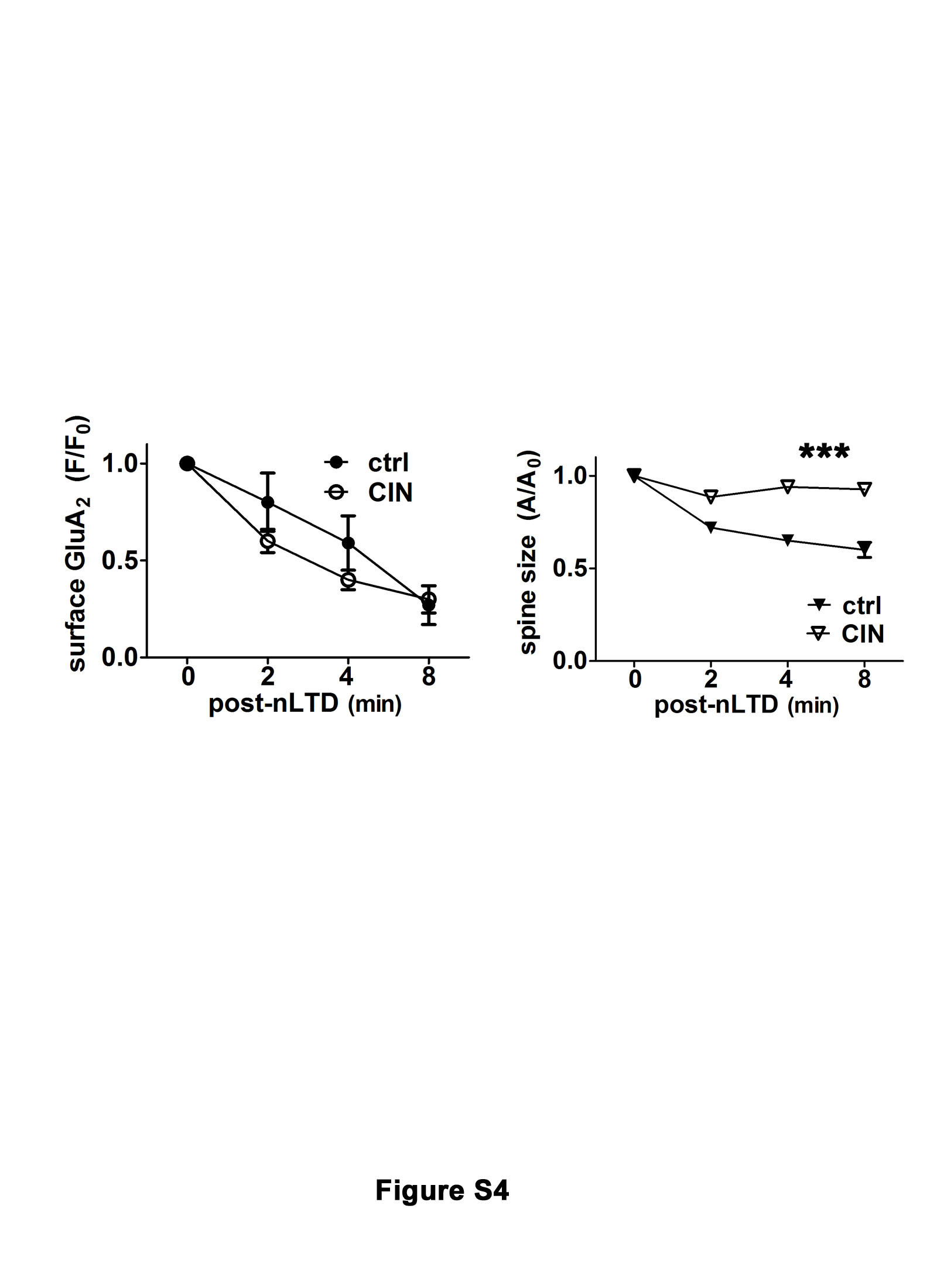

Supplement: Figure S4 — Chronophin prevents dendritic spine shrinkage but not loss of surface AMPA receptors associated with nLTD. Loss of surface AMPA receptors accompanies the spine shrinkage induced by nLTD. Changes in SEP-GluA2 fluorescence (left) and spine area (right) were quantified using time-lapse imaging during induction of nLTD for the indicated times. Control neurons display nLTD-induced loss of surface GluA2 accompanied by spine shrinkage. Chronophin (CIN) overexpression prevented dendritic spine shrinkage but had no significant effect on the loss of surface GluA2. Data are expressed as mean ± SEM; *** indicates significant difference between curves (p<0.001) using two-way ANOVA, followed by Bonferroni post hoc test; number of spines for control neurons = 59 for all time points measured; number of spines for CIN-transfected neurons = 59 for t = 0 min, 2 min, 4 min, but only 35 spines for t = 8 min, due to complete spine collapse for a subset of spines. (JPG) [file pone.0094787.s004.jpg]

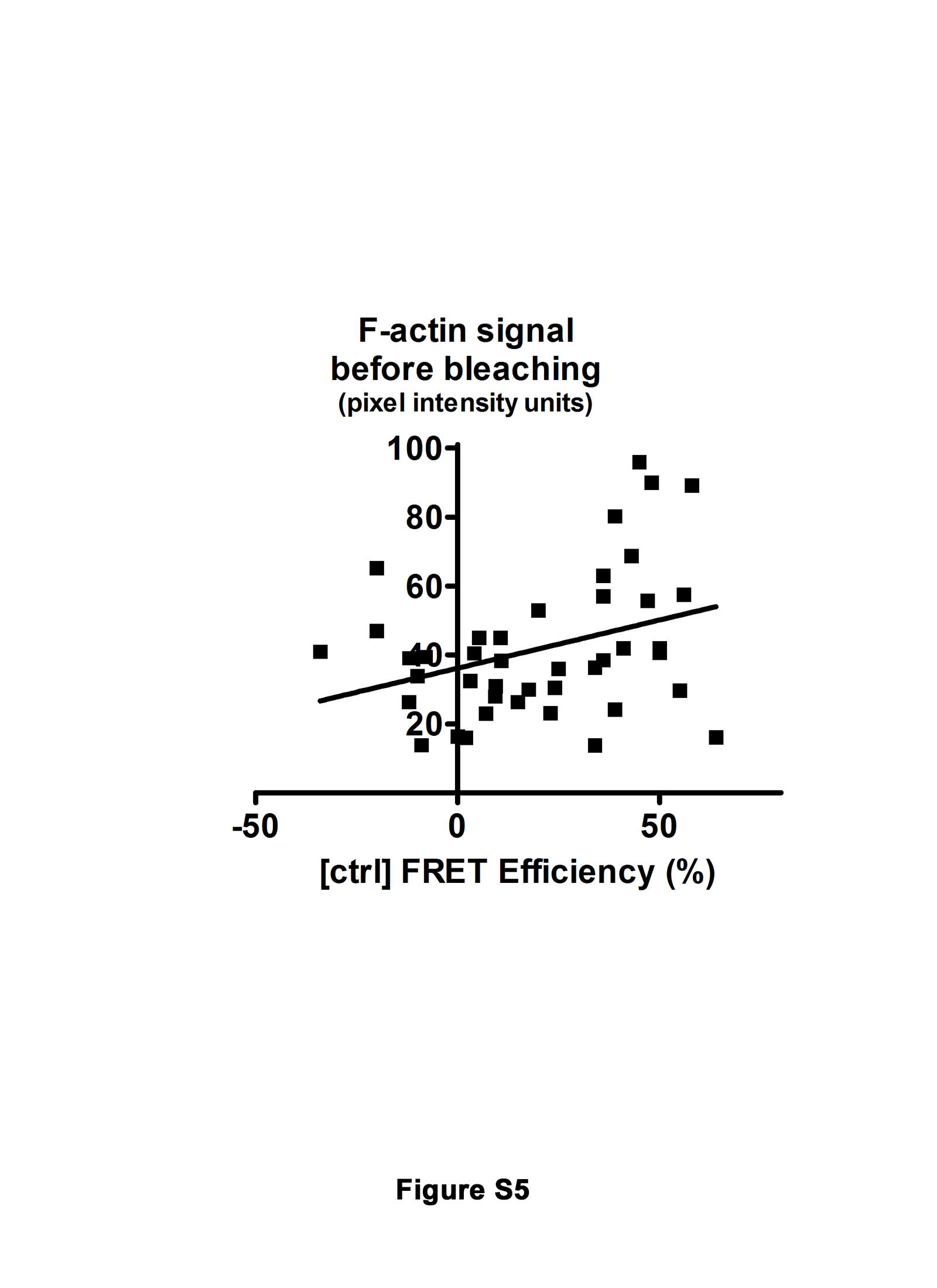

Supplement: Figure S5 — FRET efficiency is not significantly correlated with the pre-bleaching intensity of F-actin. Control (ctrl) cultures were prepared and immuno-FRET assays performed to quantify the relative molecular proximity between endogenous cofilin and endogenous F-actin within spines, as described in Methods. The graph compares the integrated intensity of the initial actin signal versus the FRET value determined using acceptor photobleaching, as described in Methods (n = 42 spines; R2 = 0.11). (JPG) [file pone.0094787.s005.jpg]

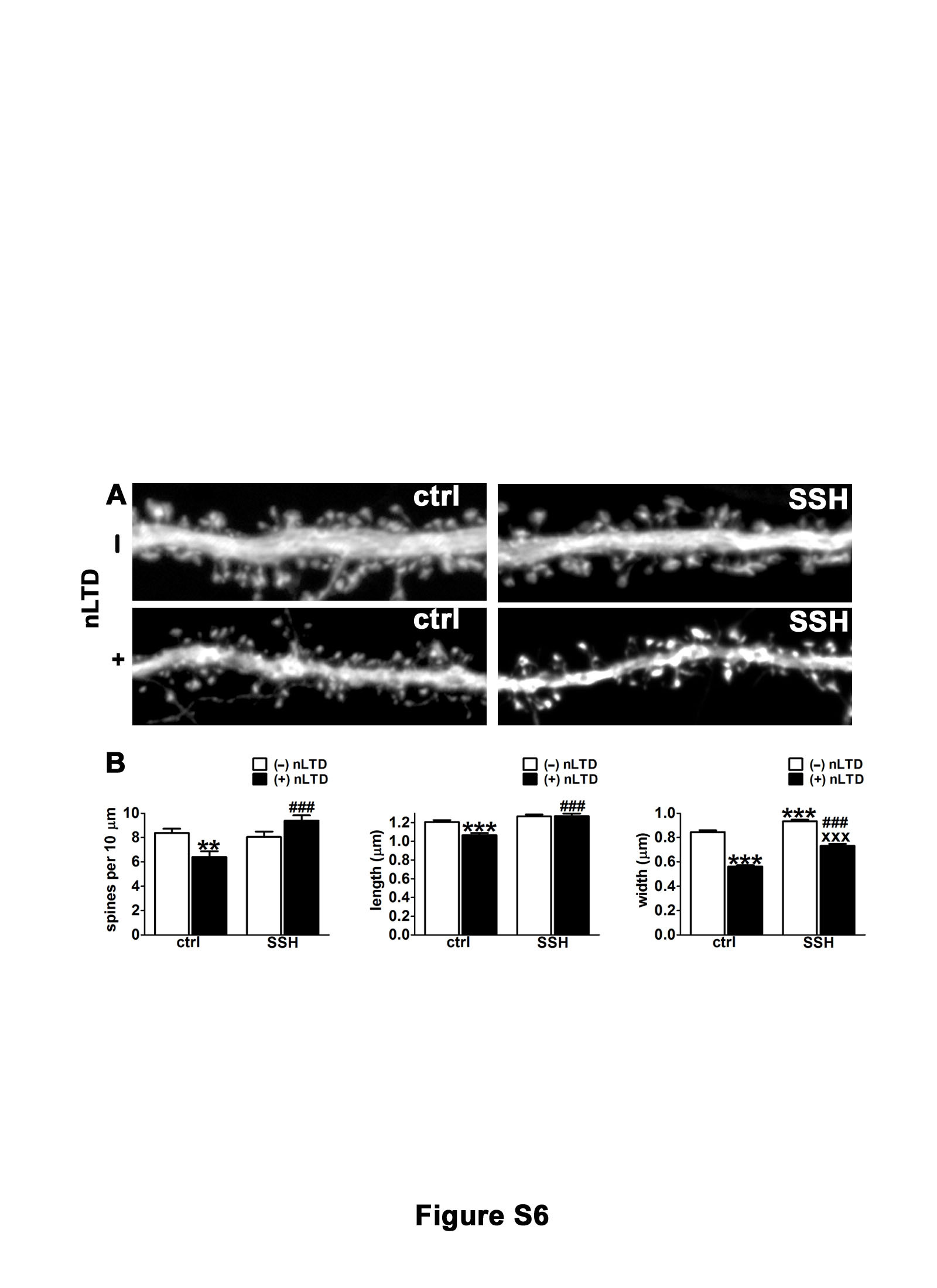

Supplement: Figure S6 — Spine loss and shrinkage induced by nLTD are attenuated by slingshot (SSH) overexpression. Neurons were cultured and transfected with eGFP as cell filler alone or together with the coflin phosphatase slingshot (SSH), as described in Methods, prior to incubation in the absence or presence of NMDA for 4 min to induce nLTD. (A) Images shown represent the eGFP channel only, for the four different treatment groups: with (+) and without (–) nLTD induction; absence or presence of SSH overexpression. nLTD causes spine loss and shrinkage in eGFP alone (ctrl) cultures (left) but not in SSH-expressing neurons (right). Image width = 27.3 µm. (B) Data are expressed as mean ± SEM; for spine density quantification, number of dendrites: ctrl = 22, nLTD (ctrl) = 30, SSH = 25, nLTD (SSH) = 26; for spine length and width quantification, number of spines: ctrl = 527, nLTD (ctrl) = 519, SSH = 578, nLTD (SSH) = 679; **p<0.01; ***p<0.001 compared to control alone; ###p<0.001 compared to nLTD alone; XXXp<0.001 compared to SSH alone, two-way ANOVA, followed by Bonferroni post hoc test. (JPG) [file pone.0094787.s006.jpg]

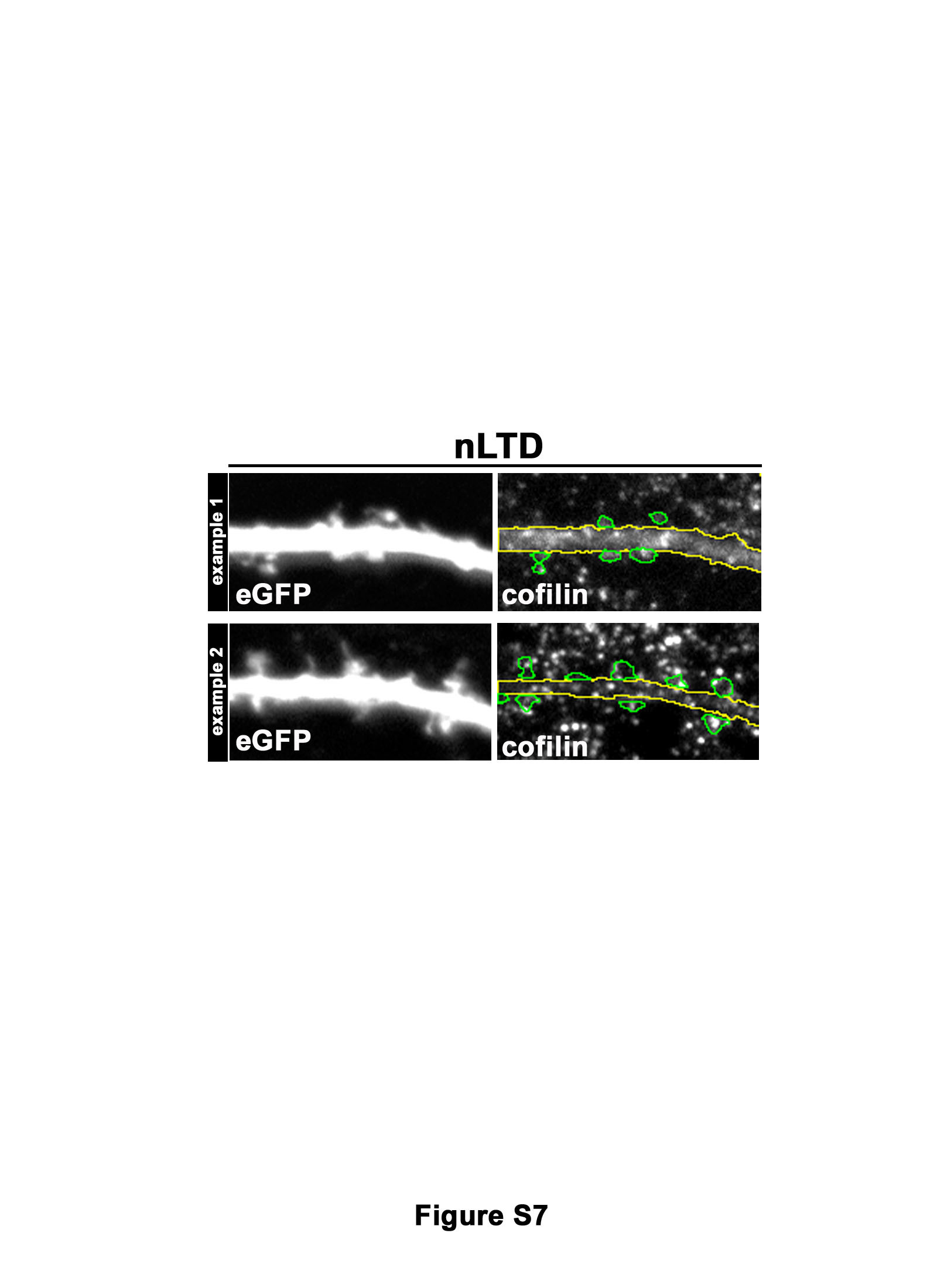

Supplement: Figure S7 — Examples of dendrites stained for endogenous cofilin 4 min post-induction of nLTD. Cofilin immunoreactivity (at right) appears weak or absent in most spines relative to control conditions (compare, e.g., to control neuron in Figure 4B2, which was processed in parallel from the same experiment, and is matched for scaling of pixel intensity). In the cofilin images (right) the yellow lines indicate the outline of the dendritic shaft created from the MAP2 image; and the green lines indicate the outlines of spines created from the eGFP cell filler image (at left), after subtraction of the MAP2 image. Image width = 18. (JPG) [file pone.0094787.s007.jpg]

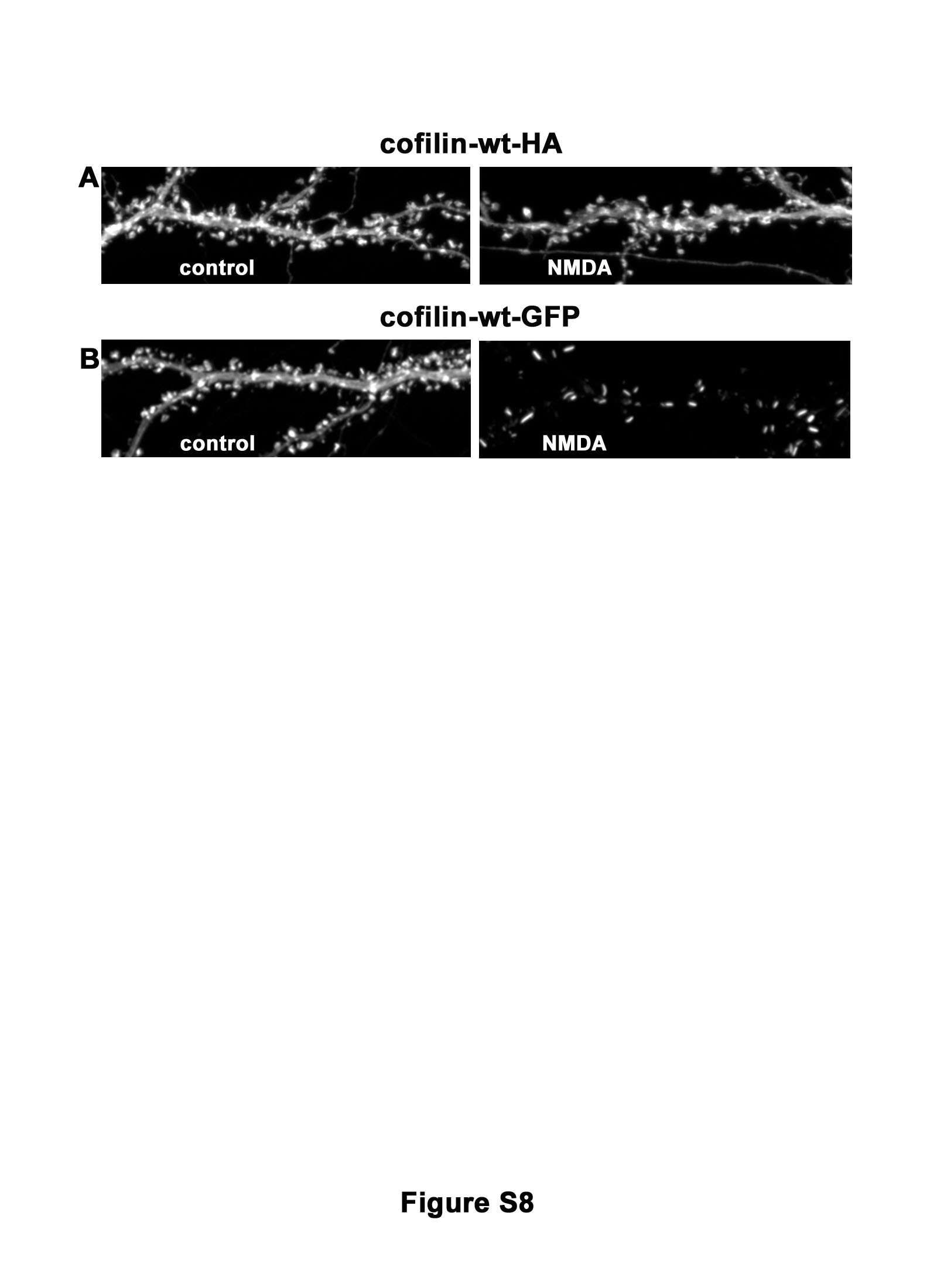

Supplement: Figure S8 — Cofilin-wt-GFP, but not cofilin-wt-HA, redistributes into aberrant aggregates following NMDA addition. Neuronal cultures were transfected with constructs to express ectopic cofilin tagged on the C-terminus with either eGFP or hemaglutinin, as indicated, incubated in the absence or presence of 40 µM NMDA for 4 min (i.e., the ‘nLTD’ condition used throughout this study), and fixed and imaged as described in Methods. HA immunoreactivity was detected using a fluorescently tagged secondary antibody. (A) Selected dendritic regions expressing cofilin-wt-HA in the absence (control) or presence of NMDA for 4 min. (B) Selected dendritic regions expressing cofilin-wt-GFP in the absence (control) or presence of NMDA. Note the extreme loss of fluorescence within the dendrite shaft and the intense accumulation of fluorescence within rod-like structures, most of which correspond to dendritic spine regions. (JPG) [file pone.0094787.s008.jpg]

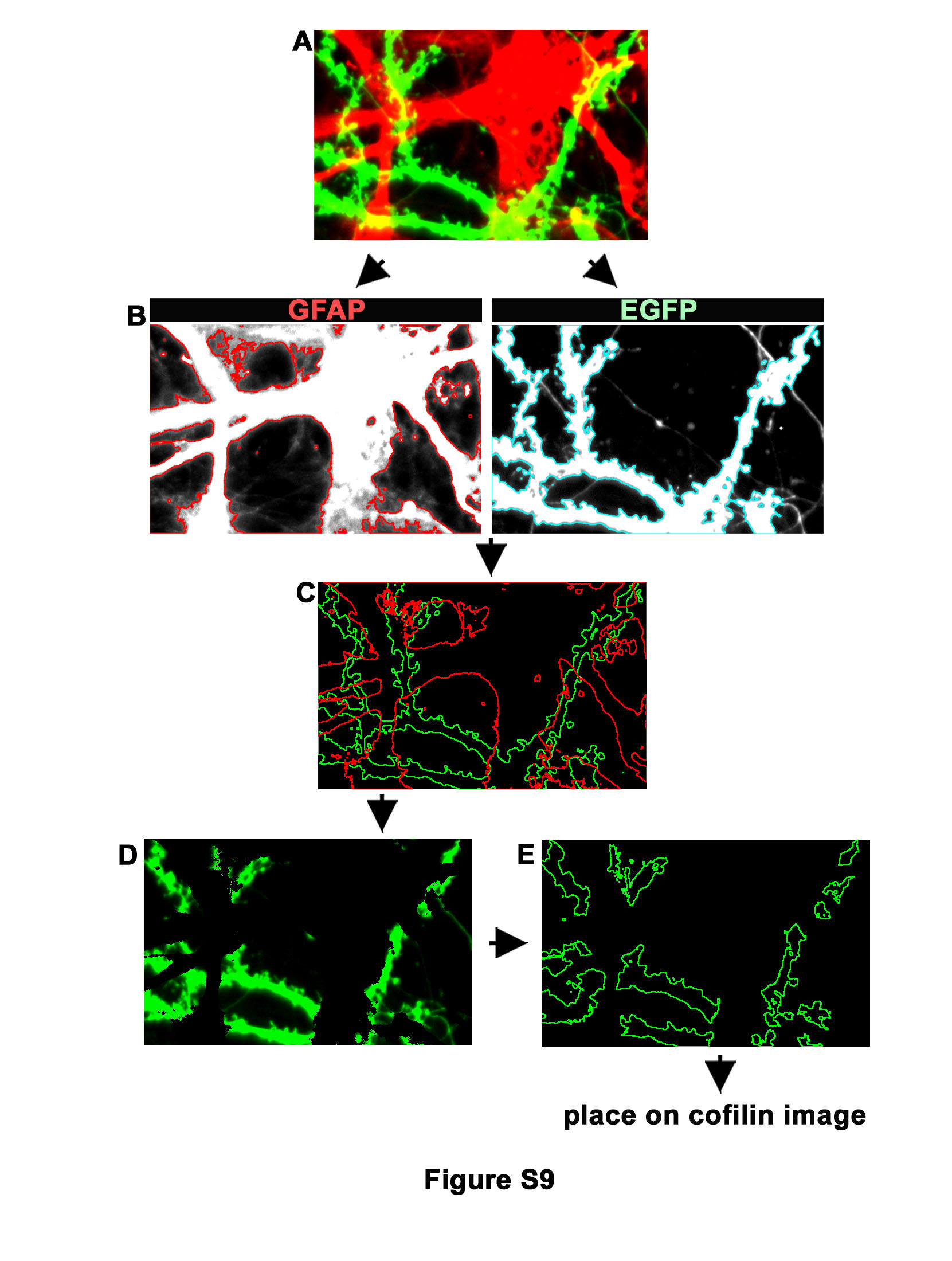

Supplement: Figure S9 — Digital processing procedure used to eliminate astrocyte regions from the dendritic region of interest prior to quantification of cofilin immunoreactivity in dendrites and spines. (A) Merged image from a region containing an eGFP expressing dendrite (green) adjacent to a GFAP-positive astrocyte (red). Note the multiple areas of spatial overlap (yellow). Image width = 52 µm. (B) In step one, binary masks are created individually from the GFAP image (left) and the eGFP image (right). (C) Next the binarized masks (shown in outline form) are digitally overlaid, and all pixels corresponding the image mask for GFAP (red outline) are subtracted from the image mask for eGFP (cyan outline), resulting in a dendritic region lacking the areas overlapping with the adjacent astrocytes (D). The corresponding outline (E) can then be overlaid onto corresponding images of interest, such as cofilin immunoreactivity, in order to quantify signals specifically in dendrites and spines that are free from significant astrocyte-associated signal. (JPG) [file pone.0094787.s009.jpg]

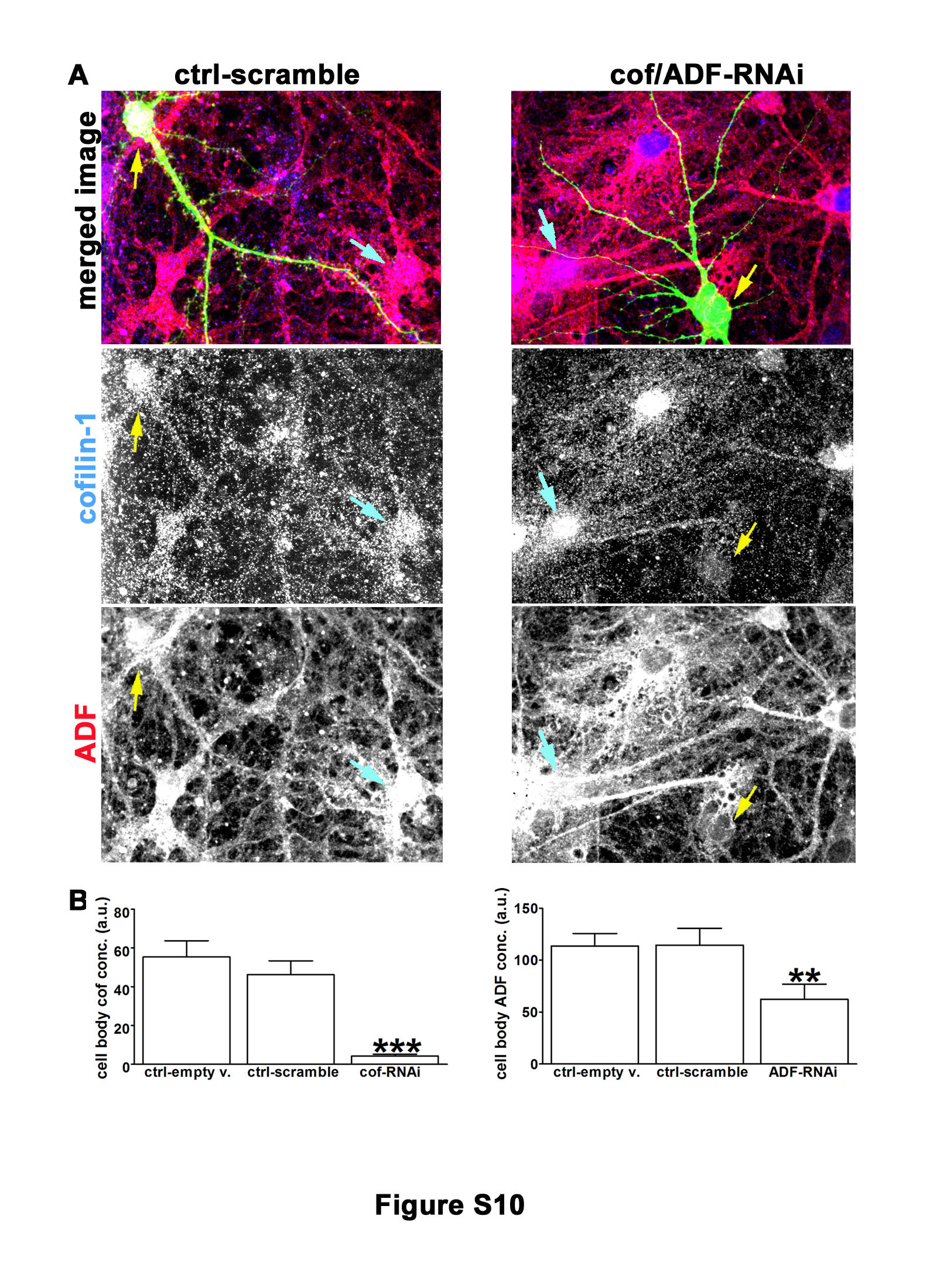

Supplement: Figure S10 — Cofilin/ADF shRNA significantly decreases both cofilin-1 and ADF levels. Neuronal cultures were prepared and shRNA-mediated silencing were carried out as described in Methods. A four day incubation with RNAi construct induced a decrease in cofilin-1 immunoreactivity by approximately 90%, and a decrease in ADF immunoreactivity by approximately 50%. Neither the empty vector nor a scrambled control sequence induced a significant change in immunoreactivity for either cofilin isoform. (A) Left: Images of a neuron expressing pSuper eGFP scrambled control shRNA, double-stained with antibodies against both cofilin 1 and ADF. Right: Images of a “knockdown neuron” expressing pSuper eGFP ADF/cofilin shRNA. The yellow arrows indicate cell bodies of transfected neurons, the cyan arrows indicate cell bodies of neighboring untransfected neurons. Image width = 127 µm. (B) Bar graphs show quantification of the relative concentration of cofilin-1 (cof) or ADF (see Methods for details of the assay). Data represent integrated intensity of the cofilin-1 or ADF signal within the cell soma, and are expressed as mean ± SEM; **p<0.01; ***p<0.001, one-way ANOVA, followed by Tukey's post hoc test; number of cells per condition: empty = 12, scramble = 9, cof/ADF-RNAi = 14. The data are expressed using arbitrary units (a.u.). (JPG) [file pone.0094787.s010.jpg]

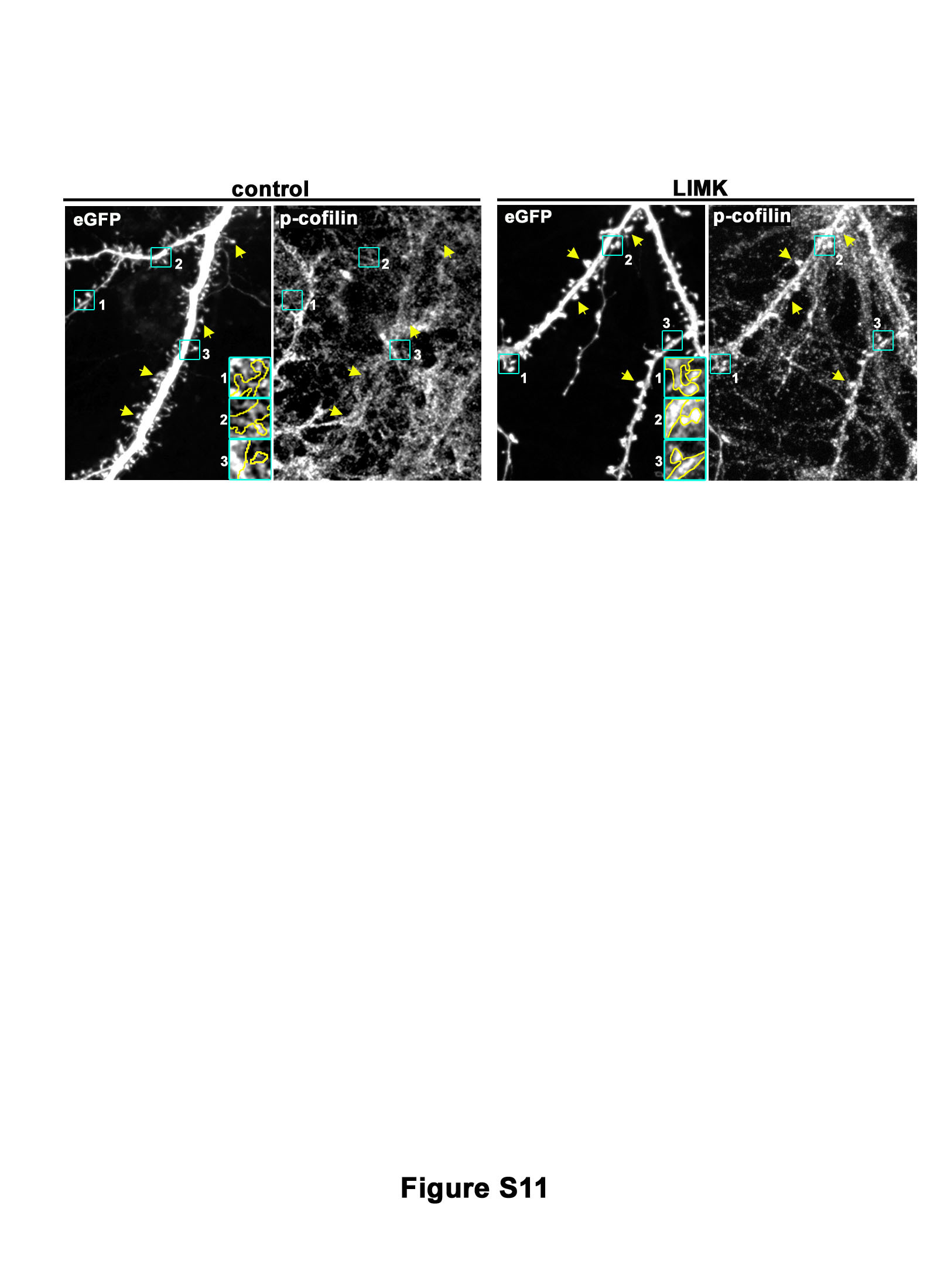

Supplement: Figure S11 — LIMK overexpression increases immunoreactivity for endogenous phospho-cofilin in neurons. Selected dendritic regions of neurons expressing either eGFP or eGFP together with LIMK, immunostained for endogenous phosphorylated cofilin (p-cofilin). Image width = 35 µm. Yellow arrows point to various spines along a region of transfected dendrite; numbered cyan square boxes refer to subregions of the dendrite shown at higher magnification in the cyan-boxed insets, in which a yellow outline of single dendritic spines is overlaid onto the image for phospho-cofilin immunoreactivity. Note that phospho-cofilin immunoreactivity is very low in control neurons (both eGFP-transfected and untransfected). Much of the immunoreactivity seen in a given field of view is attributable to astrocytes. Overexpression of LIMK (right) induces a substantial increase in immunoreactivity for phospho-cofilin in neurons, including within spines, which are now readily detectable above the signal from surrounding untransfected neurons and astrocytes. (JPG) [file pone.0094787.s011.jpg]
